# Genetic variation and phylogeography of the *Triatoma dimidiata* complex evidence a potential center of origin and recent divergence of haplogroups having differential *Trypanosoma cruzi* and DTU infections

**DOI:** 10.1371/journal.pntd.0007044

**Published:** 2019-01-28

**Authors:** Angélica Pech-May, Carlos Jesús Mazariegos-Hidalgo, Amaia Izeta-Alberdi, Sury Antonio López-Cancino, Ezequiel Tun-Ku, Keynes De la Cruz-Félix, Carlos N. Ibarra-Cerdeña, Raúl E. González Ittig, Janine M. Ramsey

**Affiliations:** 1 Instituto Nacional de Salud Pública /Centro Regional de Investigación en Salud, Tapachula, Chiapas, México; 2 Instituto Nacional de Medicina Tropical, Ministerio de Salud de la Nación, CONICET, Puerto Iguazú, Misiones, Argentina; 3 Instituto de Estudios Superiores de Chiapas, Tuxtla–Gutiérrez, Chiapas, México; 4 Universidad Anáhuac, Huixquilucan, Estado de México, México; 5 Departamento de Ecología Humana, Centro de Investigación y de Estudios Avanzados del IPN (CINVESTAV), Unidad Mérida, Yucatán, México; 6 Instituto de Diversidad y Ecología Animal (IDEA), CONICET-UNC, Facultad de Ciencias Exactas, Físicas y Naturales, Universidad Nacional de Córdoba, Córdoba, Argentina; University of Texas at El Paso, UNITED STATES

## Abstract

The population genetics of *Triatoma dimidiata* haplogroups was analyzed at landscape and sub-regional scales in Chiapas and regional level across the Mexican Neotropics, and phylogeography of the complex was re-analyzed across its complete geographic range. Two contiguous fragments of the ND4 gene were analyzed due to bias from differential haplogroup specificity using a previously designed sequence. At both landscape (anthropic modification gradient) and regional (demographic, fragmentation, biogeographic, climate) scales, lowest *T*. *dimidiata* genetic diversity occurs where there is greatest historical anthropic modification, and where *T*. *cruzi* infection prevalence is significantly highest. *Trypanosoma cruzi* prevalence was significantly higher than expected in haplogroups 1 and 3, while lower than expected in haplogroup 2. There was also a significant difference of DTUI and DTUVI infection frequencies in both haplogroups 1 and 3, while no difference of either in haplogroup 2. All haplogroups from the Mexican Neotropics had moderate to high haplotype diversity, while greatest genetic differentiation was between haplogroups 1 and 3 (above F_ST_ = 0.868, *p* < 0.0001). Divergence of the complex from the MRCA was estimated between 0.97 MYA (95% HPD interval = 0.55–1.53 MYA) and 0.85 MYA (95% HPD interval = 0.42–1.5 MYA) for ND4A and both concatenated fragments, respectively, with primary divergence from the MRCA of haplogroups 2 and 3. Effective population size for Mexican haplogroups 1 and 2 increased between 0.02 and 0.03 MYA. This study supports previous ecological niche evidence for the complex´s origin surrounding the Tehuantepec Isthmus, and provides evidence for recent divergence of three primary *dimidiata* haplogroups, with differential *T*. *cruzi* infection frequency and DTU specificity, important components of vector capacity.

## Author summary

*Triatoma dimidiata* is one of the broadest distributed triatomine species´ complexes transmitting *Trypanosoma cruzi*. In Mexico, three haplogroups of the *T*. *dimidiata* complex have been reported and all are primary vectors of Chagas disease south of the Tehuantepec Isthmus. Given their epidemiological importance, the question arises whether haplogroups have similar genetic diversity in domestic/modified landscapes, as well as infection characteristics and parasite DTU associations, key components of vector capacity. The aim of the present study was to analyze *Triatoma dimidiata* population genetics across landscapes, sub-regional, regional, and global Neotropical realm scales, using two contiguous fragments of the ND4 gene. Our results support previous evidence for the complex´s origin surrounding the Tehuantepec Isthmus, and provides evidence for recent divergence of three principal *dimidiata* haplogroups and significant secondary divergence within each. Differential *T*. *cruzi* prevalence and Discrete Typing Unit (DTU) specificity for individual haplogroups provide evidence for potential differential vector capacity within the complex in Mexico.

## Introduction

*Triatoma dimidiata* (Latreille, 1811), is one of the broadest distributed triatomine species complexes transmitting *Trypanosoma cruzi*, the etiological agent of Chagas disease. This vector complex is the only one to naturally extend throughout the northern Neotropical realm of North, Central, and South America. The complex is found in a variety of domestic, peri-urban and sylvatic habitats. In the wild, it has been found in a variety of ecotopes eg. tropical, perennial, and deciduous forest, caves, grasslands, and modified habitats [1–6]. *Triatoma dimidiata* haplogroups (Hg) are all considered primary vectors of Chagas disease south of the Tehuantepec Isthmus [1, 7–10]. The second internal transcribed spacer (ITS2) of ribosomal DNA (rDNA) provided the first genetic evidence for *T*. *dimidiata*´s complex structure, beyond original subspecies designations [11, 12]. Subsequent analyses using sequences from the cyt *b*, ND4, and 16S rRNA genes, indicated between 6% and 14% divergence among the three main haplogroups [13]. Multiple authors have now used nuclear and mitochondrial markers to analyze the complex´s population genetics, systematics, and phylogeography [4, 8, 9, 14–21]. The complex is variable for many phenotypic characteristics [22–24], including intra-domestic behavior [25], cuticular hydrocarbons [26, 27], chromosomal banding patterns [28, 29], volatile compounds [30, 31] and antennal phenotype [32]. Throughout its geographic distribution, sympatry of at least two of the haplogroups has been reported [5, 8, 16, 18, 33, 34].

In Mexico, three haplogroups of the *T*. *dimidiata* complex have been reported. Haplogroup 1 (Hg1) is reported to the east of the Tehuantepec Isthmus, originally reported from the Yucatan Peninsula (YP) and within the Palenque archeological site (northeast Chiapas) [12, 23], although it has now also been reported from northern Guatemala. In the latter case nomenclature was reverted from the original designation, and samples of Hg1 were labeled Hg3 [8, 14, 18, 20, 35]. Most recently, a proposal has been made to reassign Hg1 (original designation) as *T*. spp. aff. *dimidiata* [36]. Haplogroup 2 (Hg2) is only reported from Mexico, this Hg, transcends the Isthmus and is distributed across two Gulf of Mexico states (Tabasco, Veracruz), five states adjacent to the two former located in the high plains between the transvolcanic belt ranges (Guanajuato, San Luis Potosi, Hidalgo, Puebla, Morelos), in small pockets along the Pacific coast (Nayarit, Jalisco, Colima, Michoacan, Guerrero, Oaxaca), and recently in the YP (Campeche, Yucatán) [3, 5, 8, 10, 14–16, 24, 25]. The recent proposal designates Hg2 as *T*. *dimidiata* s.s. [36]. Haplogroup 3 (Hg3) is the predominant Hg from Mexico to Peru, although only reported in Mexico from Chiapas (east of the Tehuantepec Isthmus) [8–10, 16]. A current proposal designates this haplogroup also as *T*. *dimidiata* s.s. [36].

Chagas is the most important parasitic disease in Mexico, based on morbidity, mortality and disease burden, where an estimated 89% of the population are at risk [10, 37, 38] and disease burden continues to be ignored [39]. An estimated 13% of *T*. *cruzi* infections in Mexico are transmitted by the three *T*. *dimidiata* haplogroups [10], and together they currently transmit virtually all human infections (> 99%) east and south of the Tehuantepec Isthmus. Given their epidemiological importance, the question arises whether haplogroups have similar patterns of spatial genetic variability, as well as infection characteristics and parasite DTU specificity (key components of vector capacity) in domestic/modified or sylvatic habitats, or on regional scales. The aim of the present study was to analyze *T*. *dimidiata* population genetics at different geographic scales, since anthropic modification and demography play dominant roles in vector dynamics in the landscape, while climate and biogeographic components generally play key roles at broader geographic scales (regional, continental) [3, 10, 40]. Knowledge regarding vector and parasite dynamics, and how key indicators project at different geographic scales, is fundamental to construct effective interventions by healthcare services and vector control programs, given their operative logistics. Additionally, population-based interventions targeting practices related to vector exposure require longitudinal monitoring of local vector populations and transmission risk across landscape fragments (habitats), as well as of social development and macroeconomic policies [41]. The present study analyzes *T*. *dimidiata* haplogroups from human domestic habitats at community, sub-regional (based on demographics, ecological, and anthropic components), regional (Berriozábal county, other Mexican neotropical sites without Berriozábal), and continental scales, and in fragmented landscapes (having a gradient of anthropic modification) using the subunit 4 of the NADH dehydrogenase gene (ND4). Since variability in gene amplification success associated with specific haplogroups or certain regions, had indicated analytical bias using the currently reported ND4 fragment (650 bp) [42], the present study avoided this bias by using an additional second sequence from the ND4 gene, complementary but with no overlap to the former. Population genetics of each fragment was analyzed for all haplogroups across their distributions and at multiple geographic scales, and the phylogeography of the *dimidiata* complex is re-analyzed on a continental scale. Differential *T*. *cruzi* prevalence and Discrete Typing Unit (DTU) specificity for individual haplogroups provide evidence for potential differential vector capacity within the complex in Mexico.

## Materials and methods

**Ethics statement**: All field studies complied under Instituto Nacional de Salud Publica biosecurity regulations and were specifically included in the Biosecurity Commission approval.

### Study sites and bug collections

*Triatoma dimidiata* was collected from human communities west of the Tehuantepec Isthmus and Chimalapas forest (Nopala county on the Pacific coast, Tuxtepec and Isthmus region of Oaxaca, Veracruz, San Luis Potosí), and additionally from fragmented landscapes east of the Isthmus from northern and coastal counties across Chiapas (Berriozábal, Palenque, Tapachula, Mapastepec and the Soconusco region), and the Yucatan Peninsula (multiple sites in the Yucatán and the Zoh Laguna landscape, in southeast Campeche). Triatomine specimens were collected by inhabitants or by members of the research group (following informed consent) in the framework of community-based triatomine surveillance programs and transmission ecology analyses. Collection locations were primarily from human communities (S1 Table) or regional aggregates (Berriozábal county sub-regions, other Mexican neotropical), and from several complete landscapes, which included human communities, ecotone areas, and sylvatic conserved fragments (Ignacio Zaragoza, Nuevo Montecristo and Rio Blanco, Berriozábal; Zoh Laguna, Campeche). Partial data from the latter site have been reported previously [5], although specimens were re-analyzed with different markers herein and data are reported and included in other Mexican neotropical site and continental scale analyses.

**Fig 1:**
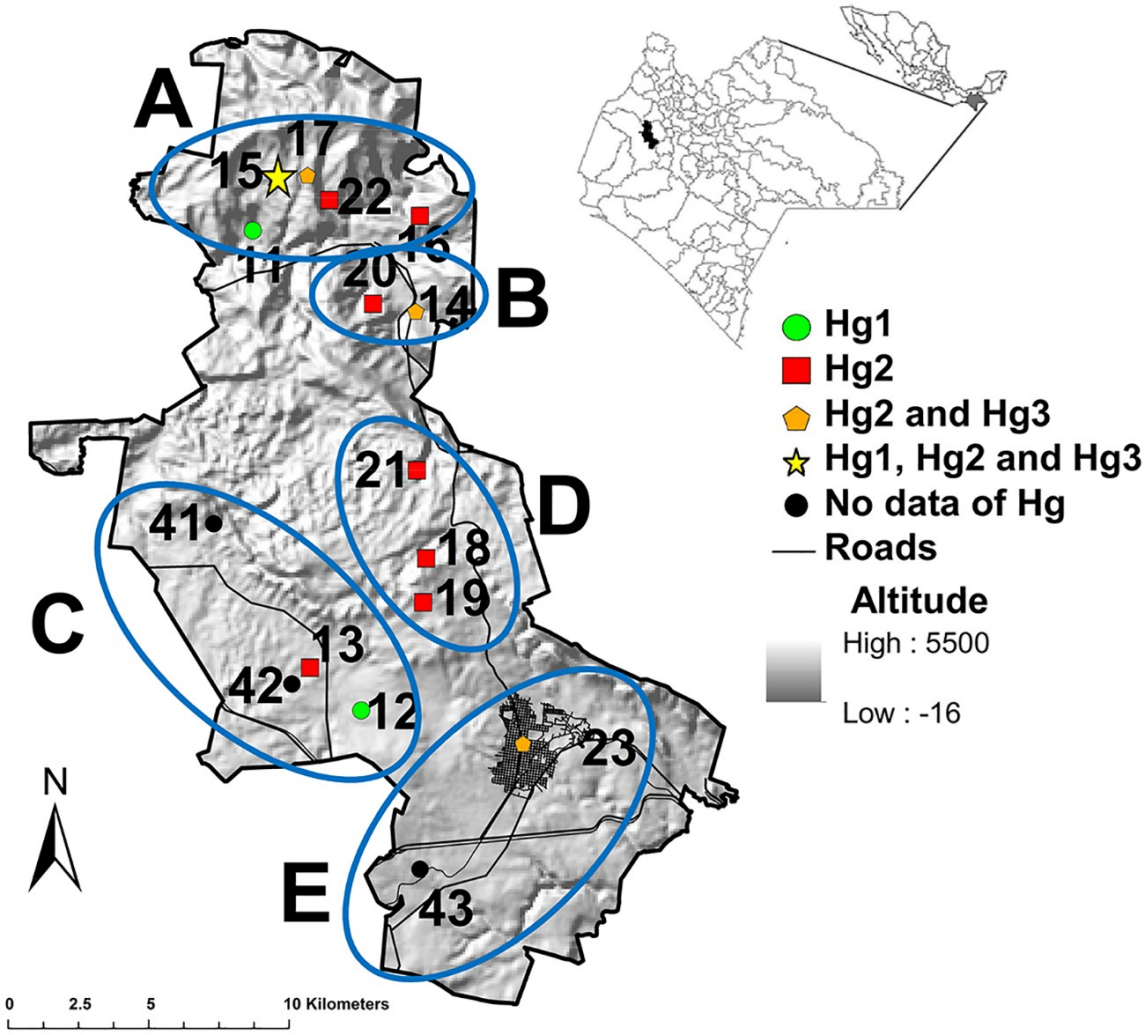
Geographic distribution of collection sites in Berriozábal sub- regiones. Map of the site of *Triatoma dimidiata* collections (S1 Table) included in analyses. The map was created using ArcGIS 10.3 (www.arcgis.com).

Berriozábal county (16°48'00" N and 93°16'22" O) in northwest Chiapas was the principal collection area for comparative genetic and *Trypanosoma cruzi* infection analyses in human communities on a regional scale (five sub-regions), and in two landscapes within two of the sub-regions. This area was chosen, based on previous *T*. *dimidiata* collections, community engagement and surveillance activities, *T*. *cruzi* infection prevalence, historical and geographic importance of the region, and failure by state health services and federal oversight, to treat and attend diagnosed cases of Chagas disease. The county has highly variable topography, with over 70% covered by the Sierra Madre Oriental mountain range in the north, and elevation ranging from 200 m to 1200 m above sea level (Fig 1). The climate is sub-humid tropical with rains ranging between 50 to 1200 mm between June and November. The vegetation is heterogeneous, principally tropical deciduous, medium sub-perennial forest, and perennial forests, with a gradient of progressive anthropic disturbance and land use change northwest to southeast. The southern sub-regions of the county concentrate large farm agriculture, livestock grazing, and small business. Communities in the county were assigned to five sub-regions, based on vegetation, landscape modification (gradient from conserved to permanent human settlement), and transport infrastructure network. Sub-region A is the most northern and conserved (proportionally) abutting the Grijalva river watershed and Malpaso dam and contains the landscape of Ignacio Zaragoza (IZ). Sub-region B is a low plain south of “A”, which contains the landscape comprised of conserved sylvatic forest, ecotone (grazing, agriculture), and two human communities (Nuevo Montecristo, Río Blanco). Sub-region C is the western region which has greatest mountain coverage abutting the Chimalapas rainforest, principally conserved, and with least human presence. Sub-region D is south of B and east of C and has the principal road running north-south, hence it is the second most modified sub-region. Sub-region E is the southernmost, historically deforested since the 17^th^ century and Spanish conquest, where the county seat is located along the highway which connects the county capital (Berriozábal) to the state capital (Tuxtla Gutierrez) and eastern Chiapas and Guatemala, and to the west, the Tehuantepec Isthmus/Chimalapas and the rest of Mexico (Fig 1). Inhabitants from all communities having over 50 houses in Berriozábal county collected bugs found inside or outside houses, placing collections in plastic bags, along with their name, date, specific collection site, and community, and these were turned over to volunteer entomology comites, before channelling to the research team. Bugs were registered and coded, taxonomically verified [43] and preserved in 90% EtOH.

The present study, as others analysing landscape modification, biotic communities [5, 44] and sociocultural components related to vector amplification along modification gradients [41], use the definition of Danielson 1991 [45] for landscape: large areas (measured at spatial scales of km^2^ or higher) that comprise more than one type of habitat distributed in numerous patches. For the purpose of landscape ecology analyses, which require specific intensive study designs, we have defined three habitats to categorize landscapes for *T*. *cruzi* dispersal: sylvatic (> 85% conserved), ecotone (partially modified, usually agriculture and livestock grazing, but not having permanent human presence), and human domestic (permanent communities) [5]. Intensive bug collections both by the population and by members of the research group (following verbal informed consent) were carried out in both rainy and dry seasons in the landscape containing the two rural communities, Nuevo Montecristo (NM) and Río Blanco (RB) (sub-region B). The landscape had been historically modified more than 70 years ago by establishment of the original community, Río Blanco. Nuevo Montecristo was recently established, as a result of the Ejército Zapatista de Liberación Nacional (EZLN) conflict, on forested land to the west side of the only road through the area and RB. The area was given to 45 families for relocation and subsistence farming by the government in 1995. Primary forest was felled and houses built central to the land grant, in accordance with agrarian reform collective holding rules. Land plots surrounding the new housing community of NM were established, further modifying the original forested area. The combined NM-RB diversity are reported in the section for sub-region B, but were also analyzed individually.

Data generated from specimens were registered in databases (collection site, date, tentative species and stages, town or georeference, county, census information, ecoregion), and were aggregated at community and landscape levels, at sub-regional, regional (Berriozábal), and other Mexican neotropical scales, to analyse haplogroup presence and abundance, and *T*. *cruzi* infection and DTU haplotype. Sampling design, size, and individual haplogroup distributions guided these analyses. More than 98% of collections were carried out by inhabitants or technical personnel in the human domestic habitat (intra or peridomestic) and hence all continental (all data from this study and registered in GenBank), regional (Berriozábal county, all other Mexican neotropical sites), and sub-regional (Berriozábal sub-regions) data are representative of this habitat category. Samples from several other regions were also collected using a representative collection design for human community aggregates (Berriozábal county, Nopala county, Yucatán state, Chiapas Soconusco coast). Finally, samples from three landscape studies are included (IZ, MC/RB, and Zoh Laguna, Campeche), since they represent a more complete survey of vector and parasite populations independent of a bias from anthropic modification.

### DNA extraction, amplification and sequencing of ND4 fragments

Genomic DNA was obtained from each sample from two legs or gonads for bug genetic analyses and from the midgut contents for *T*. *cruzi* detection and bloodmeal source identification [5]. DNA was extracted using DNAzol (Invitrogen, San Diego, California, USA), following manufacturer’s instructions, and stored at -20°C. The ND4 fragment was amplified using primers described by Dotson & Beard [42] and is designated ND4A herein. The PCR for this fragment was conducted in a final volume of 25 μl using a 50 ng DNA template, 1.5 mM MgCl_2_, buffer PCR 5X, 10 mM dNTPs, 10 pM of each primer and 0.2 U of Taq DNA polymerase (GoTaq DNA Polymerase, Promega, USA). The amplification protocol was 5 min at 94°C, followed by 36 cycles of 30 s at 94°C, 30 s at 48°C, 2 min at 72°C, followed by a 72°C extension for 7 min, and 4°C indefinitely.

Only a low proportion of samples from Mexico amplified using the ND4A (< 60%; N = 51/92), despite sample DNA quality (amplification controls). In order to test the hypothesis that low specificity was related to the population´s genetic background, and in order to develop other tools for landscape analyses, a second fragment from the ND4 gene was identified. The primers for fragment ND4B were designed from the complete genome of *T*. *dimidiata* [42] initiating at the 3´ terminal (8500–8759). Primers were designed *in silico* using the Primer3 programme (http://bioinfo.ut.ee/primer3-0.4.0/), which amplified a 230 bp fragment, 8500 F: 5´CAC AGC CCA CAA AAA CCA 3´ and 8759 R: 5´TGA CTT CCA AGG GCT CAT GT 3´. The PCR reaction was standardized for a final volume of 30 μl using a 50 ng DNA template, 2.08 mM MgCl_2_, buffer PCR 5X, 10 mM dNTPs, 10 pM of each primer and 0.16 U of Taq DNA polymerase (GoTaq DNA Polymerase, Promega, USA). The amplification protocol was 5 min at 94°C, followed by 40 cycles of 30 s at 94°C, 45 s at 50°C, 45 s at 72°C, followed by a 72°C extension for 10 min, and 4°C indefinitely. *Trypanosoma cruzi* infection and bloodmeal source were identified in all specimens using PCR as previously described [5], and parasite populations were genotyped using the mini-exon [46] and 18S rRNA genes [47, 48]. All PCR products were separated by electrophoresis in 1.5% agarose gels stained with 0.5 μg/ml ethidium bromide and visualized under UV light. PCR products were purified using the QIAquick PCR purification kit (QIAGEN, Valencia, CA) and sequencing was carried out on an ABI Prisma 310 (High-Throughput Genomics Center, University of Washington, Department of Genome Sciences) or using ABI 3730XLs (Macrogen, Korea).

Amplification success for each ND4 fragment was analysed separately and compared among haplogroups for the complete Berriozábal county, and all other Mexican Neotropical sites, using a chi-squared frequency analysis with Bonferroni correction. Significant differences in genotype success between ND4A and ND4B for each haplogroup was analysed using the log-likelihood ratio (G-test) for goodness of fit with Williams´ correction for sample size variation. All tests were considered significant if *p* < 0.05 [49].

### Genetic diversity and differentiation (F_ST_) between *T*. *dimidiata* haplogroups

Forward and reverse sequences from all samples were used to generate consensus sequences. These sequences were manually aligned using MEGA v.7 [50], and all sequences of unique haplotypes were deposited in GenBank (accession numbers ND4A: MH410755—MH410810; ND4B: S1 File). Genetic diversity was analyzed separately for both ND4A and ND4B for each haplogroup from Berriozábal, and likewise, haplogroup diversity was analyzed for all samples across the Mexican Neotropical region, without Berriozábal (S1 Table). At the landscape scale, Hg2 haplotype diversity was calculated for NM and RB separately, and for Hg2 and Hg3 from IZ (where all 3 haplogroups were collected). Genetic diversity only for Hg2 was compared among four of the five sub-regions within Berriozábal, again due to insufficient collection of the two minority haplogroups (Hg1, Hg3) in all but one of the sub-regions. It was also compared between all Berriozábal samples vs. all other Mexican neotropical samples. Genetic diversity measures were estimated from the number of mutations (*ƞ*), number of segregating sites (*S*), number of unique sites (*Su*), mean number of pairwise differences (*k*), number of haplotypes (*h*), haplotype diversity (*Hd*), nucleotide diversity (*π*) and nucleotide polymorphism index (*Ɵ*) using DNASP v.5.10 [51]. Neutrality tests *Fs* [52] and Tajima’s *D* [53] were estimated using Arlequin v.3.5 [54].

The pairwise genetic differentiation (F_ST_) between *T*. *dimidiata* haplogroups was analysed separately for both ND4A and ND4B fragments, for Berriozábal county and for all other Mexican Neotropical sites using Arlequín v.3.5. Genetic distance was estimated between haplogroups, from Berriozábal and from all other Mexican neotropical sites, using the Kimura 2-parameter (K2P) and MEGA v.7 with 10,000 random permutations.

### Phylogenetic relationship and divergence times

Phylogenetic inferences were analyzed using two datasets: a) ND4A (including, Berriozábal, all other Mexican Neotropical sites, and continental GenBank sequences), and b) concatenated ND4A and ND4B from Berriozábal and all other Mexican neotropical sites. The ND4A sequences used for the phylogeographic analysis from GenBank were: GQ202181—GQ202190;GQ202161—GQ202167; GQ202158; GQ202155; GQ202151; AF454697—AF454699; AF454695; AF454685—AF454694; JN620155—JN620169; JN620171—JN620177 [4, 18]. A median joining (MJ) haplotype network was obtained using Network v.4.6.1.1 [55] (http://www.fluxus-engineering.com) assuming epsilon of 0, to evaluate the genealogical relationships between haplogroups. Divergence times among haplogroups were assessed using Beast v.1.7 [56]. The best-fitting model of evolution for each dataset was estimated using the Bayesian Information Criterion (BIC) implemented in JModeltest v.2 [57]: a) the GTR+I+G for ND4A (Berriozábal, all other Mexican Neotropical sites from this study, and continental GenBank sequences), and b) the HKY+G model for haplotypes from concatenated sequences of ND4A + ND4B (Berriozábal and all other Mexican Neotropical sites). An uncorrelated lognormal relaxed-clock was calibrated using the nucleotide substitution rate estimated by Pfeiler et al. [58]. This mutation rate was corrected from that previously described [59], which resulted in a rate of 0.11 substitutions/site/million year (Myr). The priors for the molecular clock were normally distributed with a mean equal to 0.11 and a standard deviation of 0.011. The posterior distributions of parameters were estimated using the Coalescent Exponential Growth option for the Metropolis-coupled Markov chain Monte Carlo (MCMC) analysis. A total of 100 x 10^6^ steps sampled every 10,000 generations was used for the ND4A dataset. A total of 30 x 10^6^ steps samples every 3,000 generations were used for the concatenated sequences of ND4A + ND4B. The first 10% of trees were discarded as burn-in. The appropriate mixing of the MCMC search was analyzed using Tracer v.1.7 [60] by calculating the effective sampling sizes (ESS) for each parameter; ESS higher than 200 indicates convergence of the search. The maximum clade credibility tree was produced using TreeAnotator v.1.7 of the Beast package and visualized using FigTree v.1.4 [61]. Sequences of *T*. *phyllosoma* (access number GenBank JX848650) which belongs to the closely related *phyllosoma* complex and *T*. *nitida* (access number GenBank JX848652) belonging to the more distant *protracta* complex, were used as outgroups for phylogenetic inferences of ND4A, while sequences of *T*. *pallidipennis* (*phyllosoma* complex) generated in this study was used as outgroup also for ND4A (accession number GenBank MH410811), and for the concatenated ND4A + ND4B sequence (S1 File).

### Historical *T*. *dimidiata* demographic changes

Historical *T*. *dimidiata* population changes were inferred using the Bayesian Skyline Plot (BSP) implemented in Beast v.1.7 [56] (http://beast.bio.ed.ac.uk/). This coalescent-based demographic model uses standard MCMC sampling procedures to estimate the posterior distribution of the effective population size over time directly from heterochronous sequence data using best-fit substitution models, which were calculated using JModeltest v.2 [57]. Analyses were performed for A) ND4A from Berriozábal + other Mexican Neotropical sites + continental GenBank sequences, B) ND4A for Hg3 from CA and Colombia, C) ND4A from Berriozábal + other Mexican Neotropical sites, D) the concatenated ND4A + ND4B sequences from Berriozábal and other Mexican Neotropical sites, E) the concatenated ND4A + ND4B sequences only from Hg2 from Berriozábal and other Mexican Neotropical sites, and F) ND4A from Hg3 of Colombia [19]. Convergence to the stationary distribution and acceptable mixing were analyzed for effective sampling sizes greater than 200, using the Tracer v.1.7 [56]. The plots were obtained with Tracer v.1.7.

### *Trypanosoma cruzi* infection and bloodmeal source of *T*. *dimidiata* haplogroups

*Trypanosoma cruzi* infection prevalence, genotype success, and DTU prevalence for each *dimidiata* haplogroup was calculated for each sub-region in Berriozábal, for all Berriozábal county, for all other Mexican neotropical sites, and for the complete Mexican dataset. A Chi squared frequency analysis of independence with a Bonferroni correction was used to compare infection frequency among haplogroups. Differences between haplogroups in *T*. *cruzi* genotype success for DTUI and DTUVI were analysed using the log-likelihood ratio (G-test) with Williams´ correction for sample size variation. All tests were considered significant if *p* < 0.05 [49]. An animal:human bloodmeal index (% animal blood/ % human blood) in bugs was calculated for each haplogroup and sub-region in Berriozábal separately.

## Results

### Specificity of ND4A and ND4B fragments for *dimidiata* complex haplogroups

**Fig 2:**
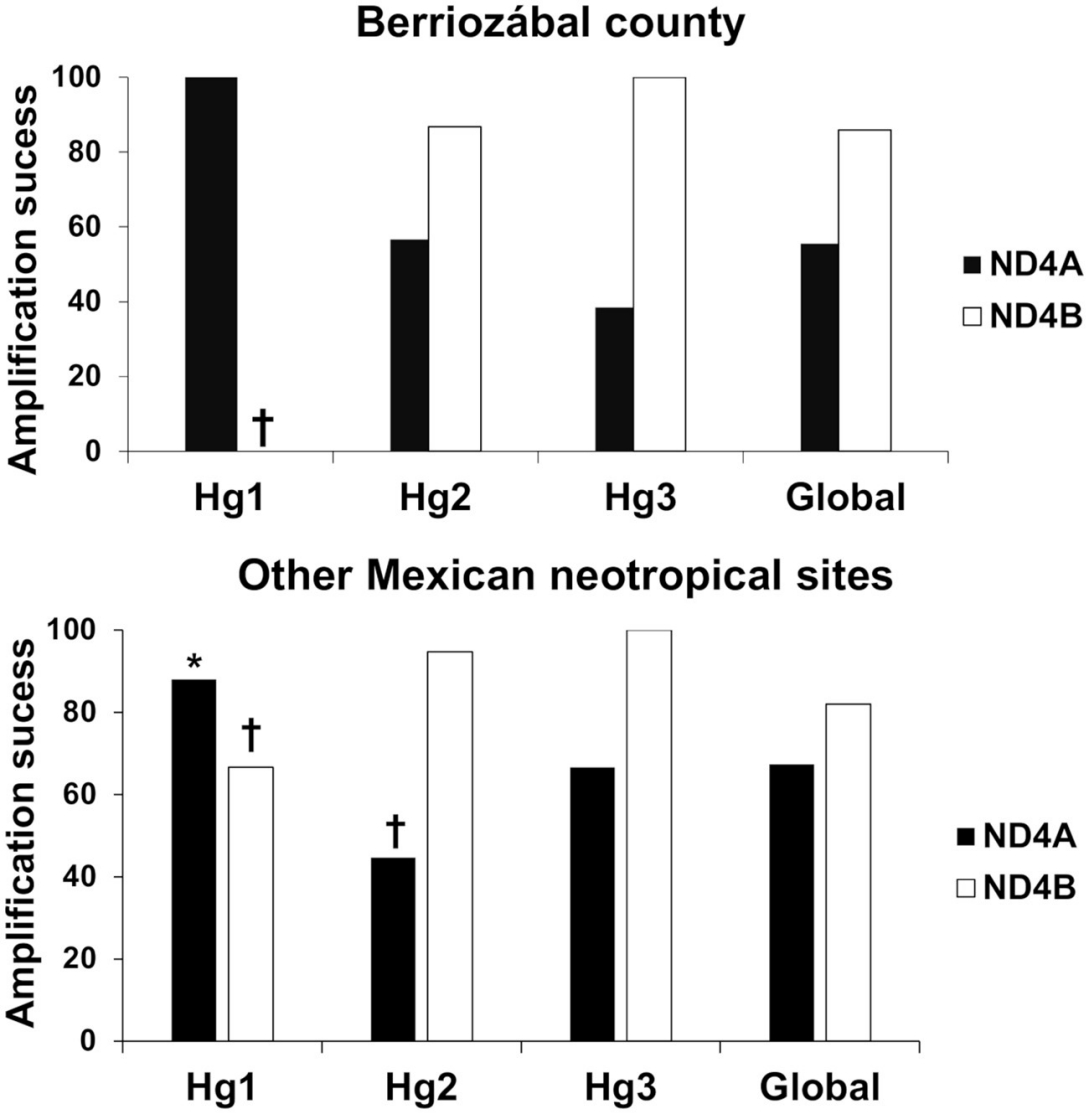
Amplification success of the three haplogroups using the ND4A and ND4B fragments.

A total of 314 specimens from Berriozábal and an additional 92 specimens from other Mexican Neotropical sites were analyzed using both ND4 fragments. Overall, only 57.3% of specimens amplified (180/314) of those collected in Berriozábal. 55.4% of all samples with quality DNA amplified using the ND4A fragment and 85.9% amplified using the ND4B. ND4A primers amplified all Hg1, but only 56.6% of Hg2, and 38.5% of Hg3 (all having quality DNA; Fig 2). In contrast, ND4B did not amplify from two specimens of Hg1(with quality DNA) from Berriozábal, although it did from 86.8% of Hg2, and 100% of Hg3. Amplification success frequency was significantly less than expected for Hg1 in Berriozábal, but this may have been due to low sample size (X^2^ = 18.2, *df* = 2, *p* < 0.00001). The difference between amplification success using ND4A and ND4B was significant for Hg2 (G = 4.9, *df* = 1, *p* = 0.02).

**Fig 3:**
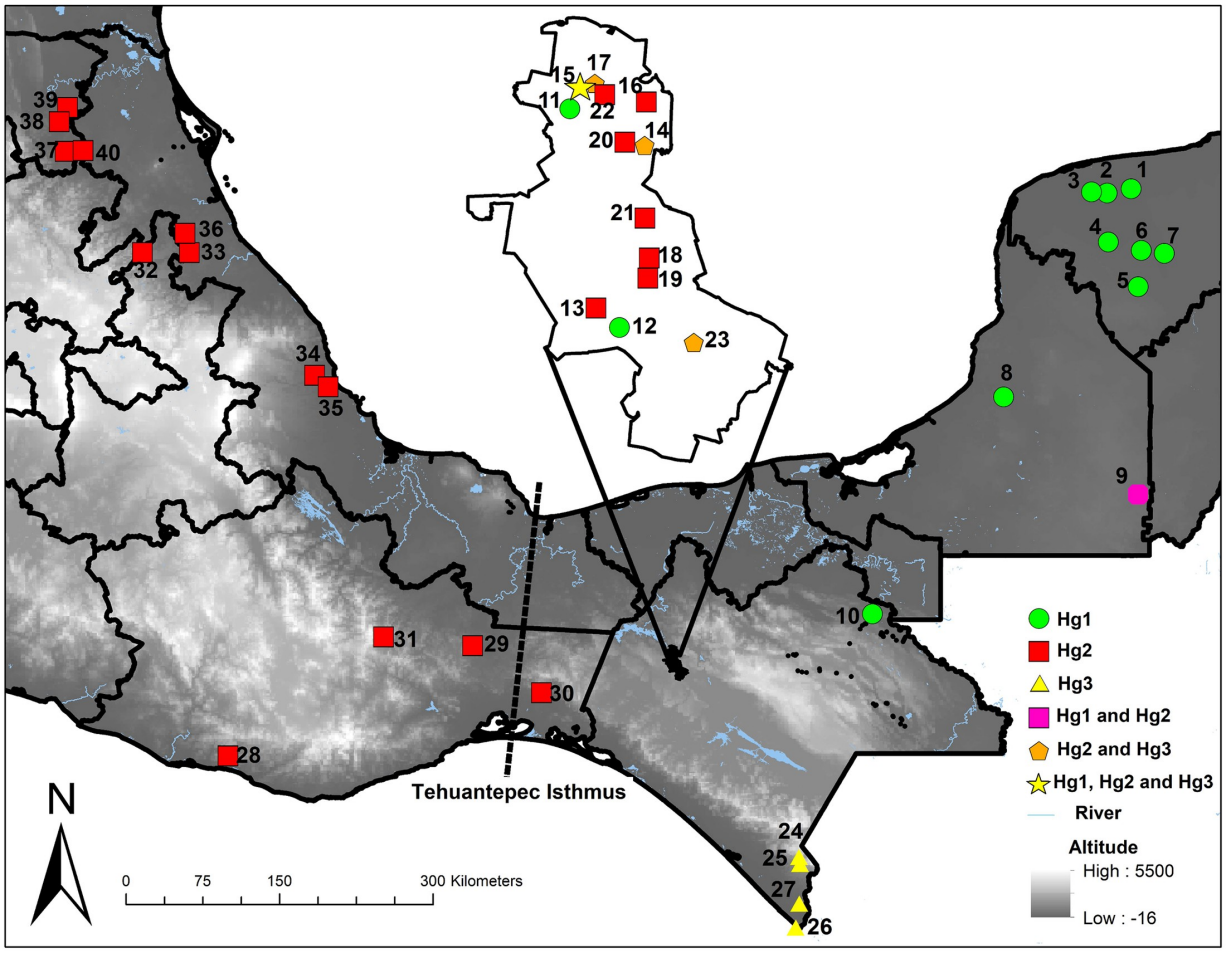
Geographic distribution of *Triatoma dimidiata* haplogroups encountered across the Mexican Neotropical region, and across the Berriozábal region in northern Chiapas (borders on the eastern edge of the Chimalapas, and the Grijalva watershed). The origin of *Triatoma dimidiata* samples included in all analyses are listed in S1 Table. The map was created using ArcGIS 10.3 (www.arcgis.com).

The amplification success rate for other Mexican Neotropical specimens was 80.2% globally, 67.4% using the ND4A and 82.0% using the ND4B. The ND4A amplified in over 88.0% of Hg1 specimens, significantly more than expected (X^2^ = 8.2, *df* = 2, *p* = 0.004), while it amplified significantly less than expected in Hg2 (44.7%, X^2^ = 8.9, *df* = 2, *p* = 0.002), and amplified 66.6% of Hg3 specimens. Using the ND4B, Hg1 were amplification significantly less than expected (X^2^ = 6.7, *df* = 2, *p* = 0.009), while 94.7% of Hg2 and 100% of Hg3 specimens amplified (Fig 2). The difference in amplification success using ND4A and ND4B was significant only for Hg2 (G = 6.9, *df* = 1, *p* = 0.008), similar to that for Berriozábal populations. The distribution of haplogroups identified from Berriozábal (Fig 1) and other Mexican neotropical sites in Mexico is summarized in Fig 3 and S1 Table.

### Comparative haplotype diversity at the landscape level

Haplotype diversity was analyzed from single landscapes in two sub-regions: A) IZ from sub-region A, and B) NM and RB in sub-region B. Ignacio Zaragoza is the only community sampled to date in Mexico where all three *dimidiata* haplogroups are sympatric (nymphs and adults). However, only one Hg1 specimen had quality DNA in IZ, while another specimen of this haplogroup was collected in another community in the same sub-region, and hence we only analyze Hg2 diversity. Hg2 from IZ had two haplotypes using ND4A (N = 3, *Hd* = 0.667 ± 0.314), and three for ND4B (N = 8, *Hd* = 0.679 ± 0.22). There was only one haplotype for Hg3 in IZ using either ND4A (N = 2) or ND4B (N = 6) fragment. Five haplotypes (N = 12) with moderate diversity for ND4A (*Hd* = 0.667 ± 0.141) were identified in Hg2 from NM, while two haplotypes (N = 13) and low diversity (*Hd* = 0.154 ± 0.126) were identified in Hg2 from RB. Using the ND4B, there were four Hg2 haplotypes (N = 13, *Hd* = 0.423 ± 0.164) in NM, and four in RB (N = 24, *Hd* = 0.236 ± 0.109). There was only one haplotype of Hg3 in NM.

### *Triatoma dimidiata* Hg2 genetic diversity among Berriozábal sub-regions

A total of 43 sequences of ND4A from *T*. *dimidiata* Hg2 were analyzed from four of the Berriozábal sub-regions: A (N = 5, 3 communities), B (N = 25, 2 communities), C (N = 6, 2 communities), and D (N = 7, 3 communities) (Table 1). There were 3.2% polymorphic sites and 10 haplotypes, varying from two to six according to sub-region. Global haplotype diversity was moderate (*Hd* = 0.482 ± 0.094), while nucleotide diversity and polymorphism index were low (*π* = 0.005 ± 0.001; *θ* = 0.008 ± 0.001, respectively). Highest genetic diversity was in sub-regions A and C, north and west in the county, located furthest from primary transportation routes and where there was the lowest proportion of modified landscape (Fig 1). The B sub-region had a significant negative neutrality test (population expansion signal) (Tajima test *D* = -1.926, *p* < 0.05) (Table 1A, S1 Fig).

**Table 1:** Genetic diversity indices for *Triatoma dimidiata* Hg2 from Berriozábal sub-regions using fragments ND4A and ND4B.

Indices	ND4A					ND4B				
	Sub-region A	Sub-region B	Sub-region C	Sub-region D	Global	Sub-region A	Sub-region B	Sub-region C	Sub-region D	Global
N	5	25	6	7	43	11	37	6	12	66
*η*	12	11	11	8	17	5	8	6	6	14
*S*	12	11	10	8	16	5	8	6	6	14
*SU*	494	495	496	498	490	153	150	152	152	144
*k*	6.8	1.24	3.6	2.286	2.636	2.618	0.781	2	1.773	1.727
*h*	4	6	3	2	10	3	5	4	5	11
*Hd* ± de	0.900±0.161	0.427±0.122	0.600±0.215	0.286±0.196	0.482±0.094	0.691±0.086	0.297±0.095	0.800±0.172	0.758±0.093	0.58±0.068
*π* ± de	0.013±0.002	0.002±0.0009	0.007±0.003	0.004±0.003	0.005±0.001	0.016±0.002	0.004± 0.001	0.012±0.005	0.011±0.003	0.01±0.004
*θ* ± de	0.011±0.003	0.006±0.001	0.009±0.002	0.006±0.002	0.008±0.001	0.01±0.004	0.012±0.004	0.016±0.006	0.012± 0.005	0.018± 0.004
Fu´s test *Fs*	-	-1.267	-	-	1.389	2.913	-1.066	-	-0.505	0.21
Tajima´s Test *D*	-	-1.926*	-	-	-0.912	2.044*	-1.718	-	-0.41	-1.198

*N*: number of sequences; *η*: number of mutations; *S*: number of segregating sites; *Su*: number of unique sites; *k*: mean number of pairwise differences; *h*: number of haplotypes; *Hd*: haplotype diversity; *π*: nucleotide diversity; *θ*: nucleotide polymorphism index. SD: standard deviation. Fu (1996) *Fs* indice and Tajima´s *D* (Tajima 1989). Statistical significance * *p* < 0.05.

A total of 66 sequences of ND4B were analyzed from Hg2 from the sub-regions A (N = 11), B (N = 37), C (N = 6), and D (N = 12). There were 8.9% polymorphic sites and 11 haplotypes (ranging between 3 and 5 in each sub-region). Global ND4B haplotype diversity was moderate (*Hd* = 0.584 ± 0.070), but nucleotide diversity and polymorphism index were low (*π* = 0.010 ± 0.001; *θ* = 0.018 ± 0.005, respectively). The same sub-regions with greatest genetic diversity for the ND4A fragment, A and C, were also the highest using the ND4B. A significant positive neutrality test (bottleneck signal) was identified in the A sub-region only (Table 1B, S1 Fig).

### Regional haplogroup genetic diversity in Berriozábal and in other Mexican neotropical sites

A total of 51 sequences were obtained for ND4A from Berriozábal county (Table 2A). There were 12.8% polymorphic sites and 13 haplotypes with moderate haplotype diversity (*Hd* = 0.624 ± 0.077), although nucleotide diversity and polymorphism index were low (*π* = 0.024 ± 0.005; *θ* = 0.032 ± 0.003, respectively). Most samples were identified as Hg2 (N = 43, 10 haplotypes), while specimens of the other two haplogroups were also present (Hg1: N = 3, 2 haplotypes; Hg3: N = 5, 1 haplotype). None of the neutrality tests were significant. The ND4B fragment was analyzed using 79 sequences from across Berriozábal. There were 19.0% polymorphic sites and 13 haplotypes, with above moderate diversity (*Hd* = 0.686 ± 0.051), while nucleotide diversity and polymorphism index were low (*π* = 0.039 ± 0.005; *θ* = 0.039 ± 0.007, respectively). Once again, most specimens were identified as Hg2 (N = 66, 11 haplotypes), along with only Hg3 (N = 13, 2 haplotypes), since the ND4B did not amplify from Hg1 samples. None of the neutrality tests were significant.

**Table 2:** Genetic diversity indices for *Triatoma dimidiata* haplogroups using the fragments ND4A and ND4B.

Fragment	Indices	Berriozábal county				Other Mexican neotropical sites			
		Hg1	Hg2	Hg3	Global	Hg1	Hg2	Hg3	Global
**ND4A 506 bp**	N	3	43	5	51	37	17	6	60
	*η*	7	17	0	73	46	27	12	113
	*S*	7	16	0	65	42	25	12	99
	*SU*	499	490	506	441	464	481	494	407
	*k*	4.667	2.636	0	12.355	3.878	5.574	4.4	27.58
	*h*	2	10	1	13	26	12	5	43
	*Hd* ± de	0.667±0.314	0.482±0.094	0	0.624±0.077	0.916±0.042	0.941±0.043	0.933±0.122	0.963±0.017
	*π* ± de	0.009±0.004	0.005±0.001	0	0.024±0.005	0.007±0.001	0.011±0.002	0.008±0.002	0.054±0.004
	*θ* ± de	0.009±0.003	0.008±0.001	0	0.032±0.003	0.019±0.003	0.014±0.002	0.01±0.003	0.041±0.004
	Fu´s test *Fs*	-	-1.123	-	0.586	-21.112***	-3.22733	-	-8.30504
	Tajima´s Test *D*	-	-0.912	-	-0.505	-2.19317**	-0.99154	-	1.04085
**ND4B 158 bp**	N	-	66	13	79	28	36	9	73
	*η*	-	14	2	31	9	10	7	28
	*S*	-	14	2	30	9	10	7	28
	*SU*	-	144	156	128	149	148	151	130
	*k*	-	1.727	0.308	6.241	1.455	1.538	3.833	6.629
	*h*	-	11	2	13	9	9	4	22
	*Hd* ± de	-	0.580±0.068	0.154±0.126	0.686±0.051	0.810±0.058	0.614±0.089	0.750±0.112	0.877±0.028
	*π* ± de	-	0.01±0.004	0.001±0.001	0.039±0.005	0.009±0.001	0.009±0.002	0.024±0.003	0.041±0.002
	*θ* ± de	-	0.018±0.004	0.004±0.002	0.039±0.007	0.014±0.004	0.015±0.004	0.016±0.006	0.036±0.006
	Fu´s test *Fs*	-	-2.899	-	-1.268	-3.699**	-2.84372	-	-1.54934
	Tajima´s Test *D*	-	-1.198	-	0.086	-1.17	-1.10332	-	0.47351

*N*: number of sequences; *η*: number of mutations; *S*: number of segregating sites; *Su*: number of unique sites; *k*: mean number of pairwise differences; *h*: number of haplotypes; *Hd*: haplotype diversity; *π*: nucleotide diversity; *θ*: nucleotide polymorphism index. SD: standard deviation. Fu (1996) *Fs* indice and Tajima´s *D* (Tajima 1989). Statistical significance ***p < 0.0001, **p < 0.02.

The complete Mexican neotropical dataset, without Berriozábal, for ND4A included 60 sequences. There were 19.6% polymorphic sites and 43 haplotypes, which varied from 5 to 26, depending on the haplogroup. Haplotype diversity was high (*Hd* = 0.963 ± 0.017), while nucleotide diversity and polymorphism index were moderate (*π* = 0.054 ± 0.004; *θ* = 0.041 ± 0.004, respectively). Only Hg1 had significant negative neutrality tests, indicating a population expansion signal (*Fs* = -21.112; and Tajima *D* = -2.193). Analyses of the ND4B fragment for other Mexican neotropical specimens included 73 sequences (Hg1 = 28, Hg2 = 36, Hg3 = 9) (Table 2B). There were 17.7% polymorphic sites and 22 haplotypes, which varied from four to nine. Haplotype diversity was high (*Hd* = 0.877 ± 0.028), while the nucleotide diversity and polymorphic index were moderate (*π* = 0.041 ± 0.002, *θ* = 0.036 ± 0.006, respectively). Once again, all neutrality tests were not significant for the global set, while they were significant and negative for Fu´s for Hg1 only (*Fs* = -3.699), indicating a population expansion signal.

### Population differentiation and genetic distances among haplogroups

A high genetic differentiation was observed among *T*. *dimidiata* haplogroups from Berriozábal, with global F_ST_ values of 0.936 (*p* < 0.0001) and 0.918 (*p* < 0.0001), for ND4A and ND4B, respectively. Greatest genetic differentiation occurred between Hg1 and Hg3 (ND4A), with 0.09 ± 0.012 K2P genetic distance (Table 3). Likewise, global genetic differentiation observed among all other Mexican neotropical sites was high, 0.913 (*p* < 0.0001) and 0.828 (*p* < 0.0001), for ND4A and ND4B, respectively. Greatest genetic difference was again between Hg1 and Hg3, for both ND4A and ND4B, with 0.103 ± 0.015 and 0.085 ± 0.024 K2P genetic distance, respectively.

**Table 3:** Pairwise genetic differentiation (F_ST_)/K2P genetic distance between *T*. *dimidiata* haplogroups. Below diagonal is for the ND4A fragment, and above the diagonal is for the ND4B fragment. Bold is for the three highest values.

	Berriozábal			Other Mexican neotropical sites		
	Haplogroup 1	Haplogroup 2	Haplogroup 3	Haplogroup 1	Haplogroup 2	Haplogroup 3
**Haplogroup 1**			-		0.838***/0.05	0.868***/0.085
**Haplogroup 2**	0.944***/0.09		0.918***/0.122	0.915***/0.095		0.786***/0.046
**Haplogroup 3**	0.965***/ 0.09	0.928***/0.066		0.928***/0.103	0.847***/0.059	

Statistical significance ***p < 0.0001.

### Phylogenetic relationship and divergence times: Continental analysis using the ND4A fragment

**Fig 4:**
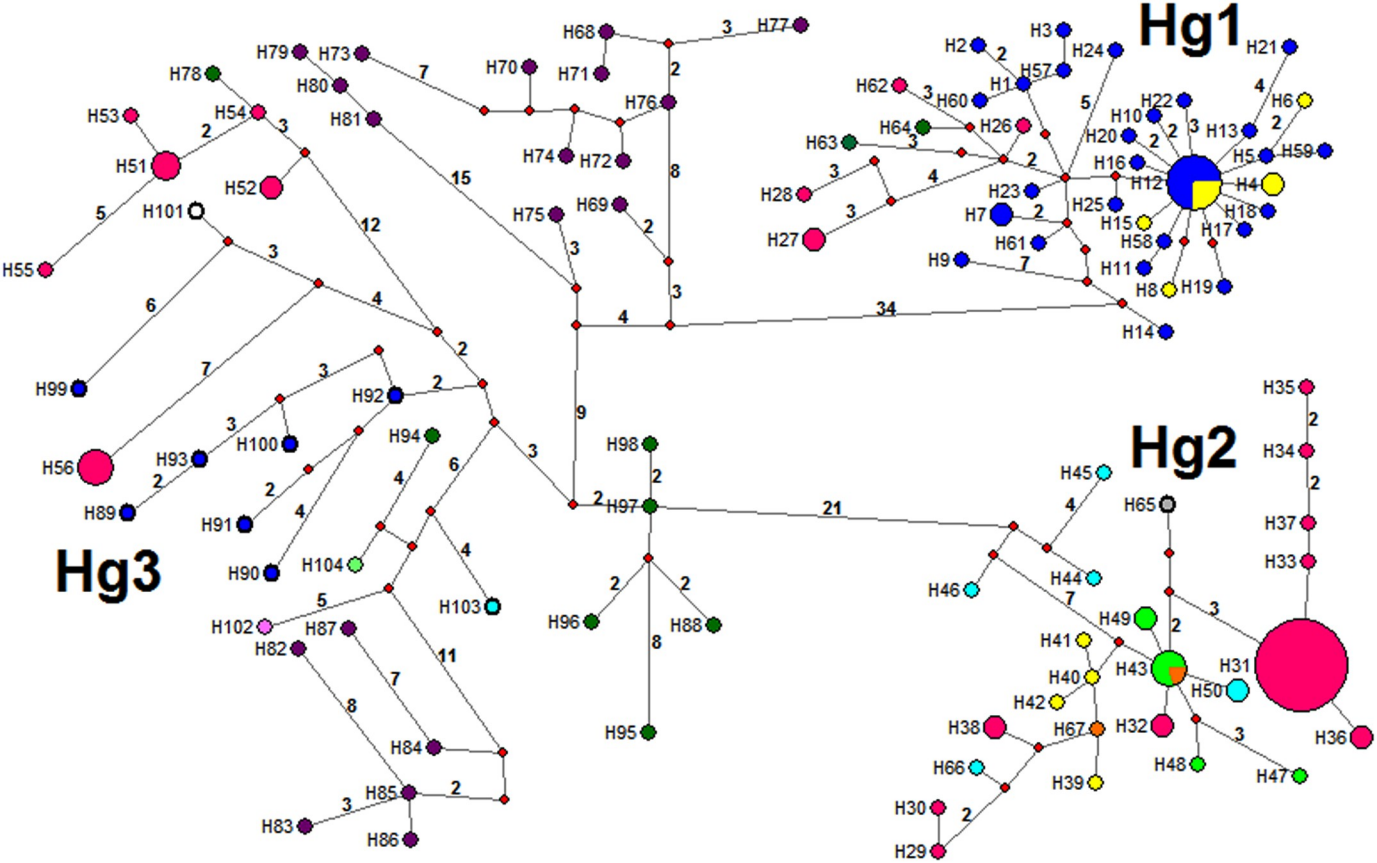
Median-joining haplotype network for the *T*. *dimidiata* complex, based on 506 nucleotides of ND4A (Neotropical region in Mexico + GenBank samples across the continent). Haplotype frequency is represented by the size of nodes and missing haplotypes are shown as red circles. The line connecting haplotypes represents one mutational step, whereas numbers along the lines are the total number of mutational steps. Colours indicate: yellow = Campeche; green = San Luis Potosí; blue = Yucatán; turquoise = Oaxaca; orange = Hidalgo; fuchsia = Chiapas; gray = Veracruz; green dark = Guatemala; White with black line = El Salvador; blue with black line = Honduras; pink = Costa Rica; turquoise with black line = Panamá; purple = Colombia; green lime = Ecuador.

The minimum spanning network using 104 unique haplotypes, including all new sequences reported herein, and those registered in Genbank, evidence three principal haplogroups separated by 23–73 mutational steps. The Hg1contains Mexican samples and two haplotypes (H63 and H64) from Yaxhá (Petén, Guatemala), which are 3 to 5 mutations from H26 and H62 sequences (Palenque, Mexico), and between 9 and 12 mutations from Berriozábal (H27 and H28, Ignacio Zaragoza, Benito Juárez, and el Sabinito) (Fig 4). The Hg1 (H14) is at least 40 mutations from Colombian Hg3 (H69). Mexican haplotypes from Hg3 from the Pacific coast (H51—H55) are one mutation from H78 (Alta Verapaz, Guatemala), while the Berriozábal specimens belonged to one haplotype (H56) 11 to 16 mutations from H101 (El Farito, Salvador) and H90 (Tegucigalpa, Honduras), respectively. Guatemala and other CA Hg3 haplotypes are at least 23 mutations from the Mexican Pacific coast sub-clade of Hg2 (H44, H46), and an additional 8 mutations from the remaining principal Hg2 sub-clade west and north of the Tehuantepec Isthmus (Fig 4, S1 Table).

**Fig 5:**
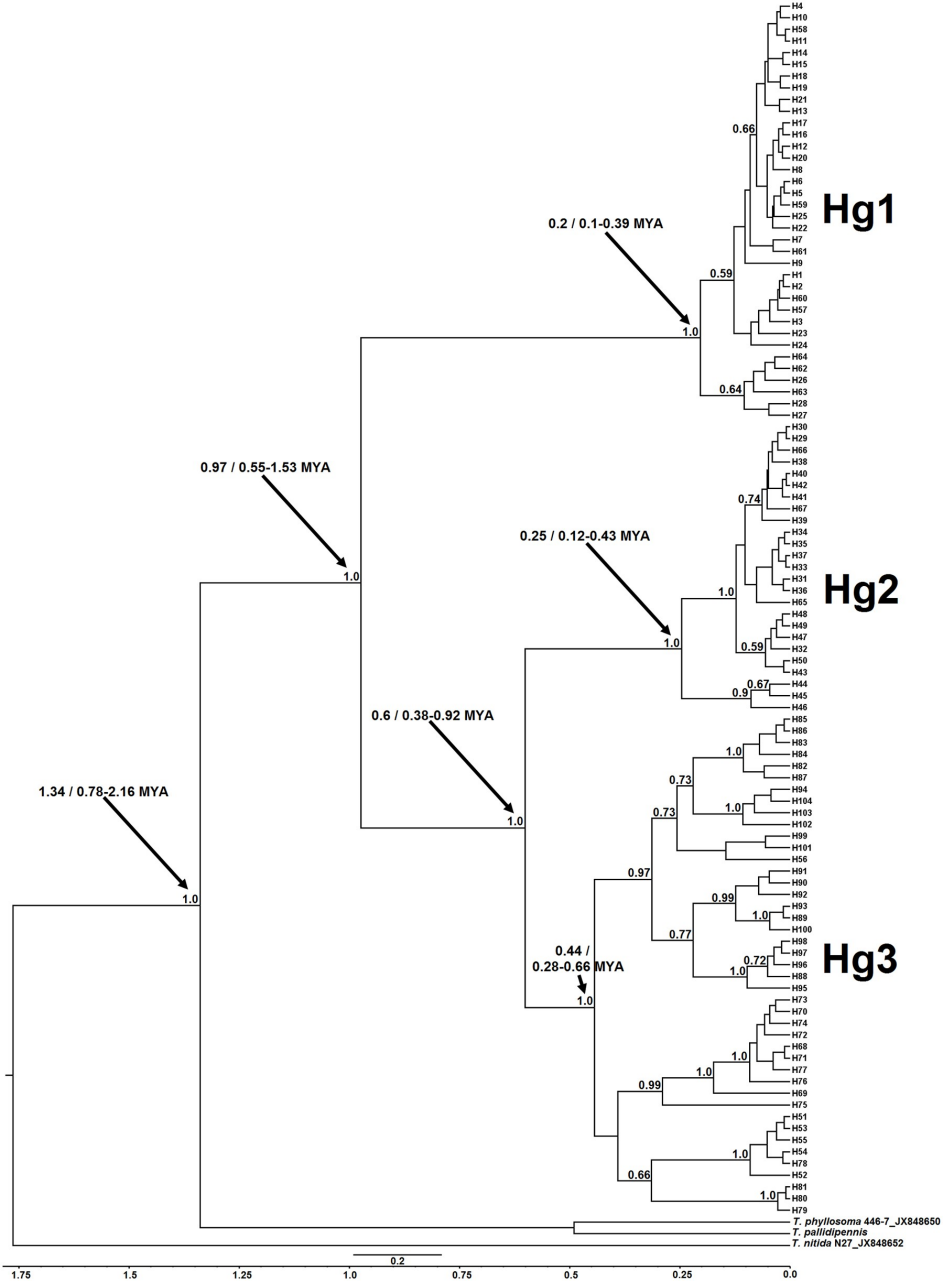
Calibrated maximum-clade-credibility tree for the *T*. *dimidiata* complex using the ND4A fragment. Numbers above each branch represent posterior probabilities PP ≥ 0.95. *T*. *pallidipennis*, *T*. *phyllosoma* and *T*. *nitida* were used as outgroup. The scale bar represents the expected number of nucleotide substitutions per site and below the line, time in million years ago (MYA) and the numbers indicated by arrows are the time estimate and the 95% HPD.

The divergence time analysis of 104 unique haplotypes from continental ND4A sequences used the GTR+I+G model (-InL = 3026.6304, Delta BIC = 0, BIC = 6719.2609) with p-inv = 0.5260 and gamma = 0.999. The *T*. *dimidiata* complex phylogenetic tree has two main clades with 1.0 PP; clade 1 contains Hg1 haplotypes, while the second clade contains both Hg2 and Hg3. The Hg1 grouped samples from three Mexican States (Yucatan, Campeche, Chiapas) and Yaxhá, Guatemala. The Hg2 grouped samples only from Mexico: Campeche, Chiapas (Berriozábal), Veracruz, Isthmus, Pacific coast and northern Oaxaca, Hidalgo, and San Luis Potosí. The Hg3 has the broadest distribution with samples from the high plain and coast of Chiapas (México), Santander, Guajira, Boyaca, Cesar (Colombia), Alta Verapaz (Guatemala), The Cade (Ecuador), San José (Costa Rica), Santa Fe (Panamá), Carrizalón, Tegucigalpa, and Yoro (Honduras), and Farito (El Salvador) (Fig 5, S1 Table).

Analyses using a relaxed molecular clock for ND4A estimated the MRCA of the *T*. *dimidiata* complex at 0.97 million years ago (MYA) (95% HPD interval = 0.55–1.53 MYA). Divergence of Hg2 and Hg3 was estimated earlier than for Hg1, at 0.60 MYA (95% HPD interval = 0.38–0.92 MYA). The MRCA for Hg3 divergence in to the two subclades A and B, was estimated at 0.44 MYA (95% HPD interval = 0.28–0.66 MYA), while divergence of the primary Gulf and secondary Pacific coast clades of Hg2 was estimated more recently, similar to that for Hg1, at 0.25 MYA (95% HPD interval = 0.12–0.43 MYA). The MRCA for Hg1 was estimated at 0.20 MYA (95% HPD interval = 0.10–0.39 MYA) (Fig 5).

### Phylogenetic relationship and divergence times using the concatenated ND4A and ND4B fragments

**Fig 6:**
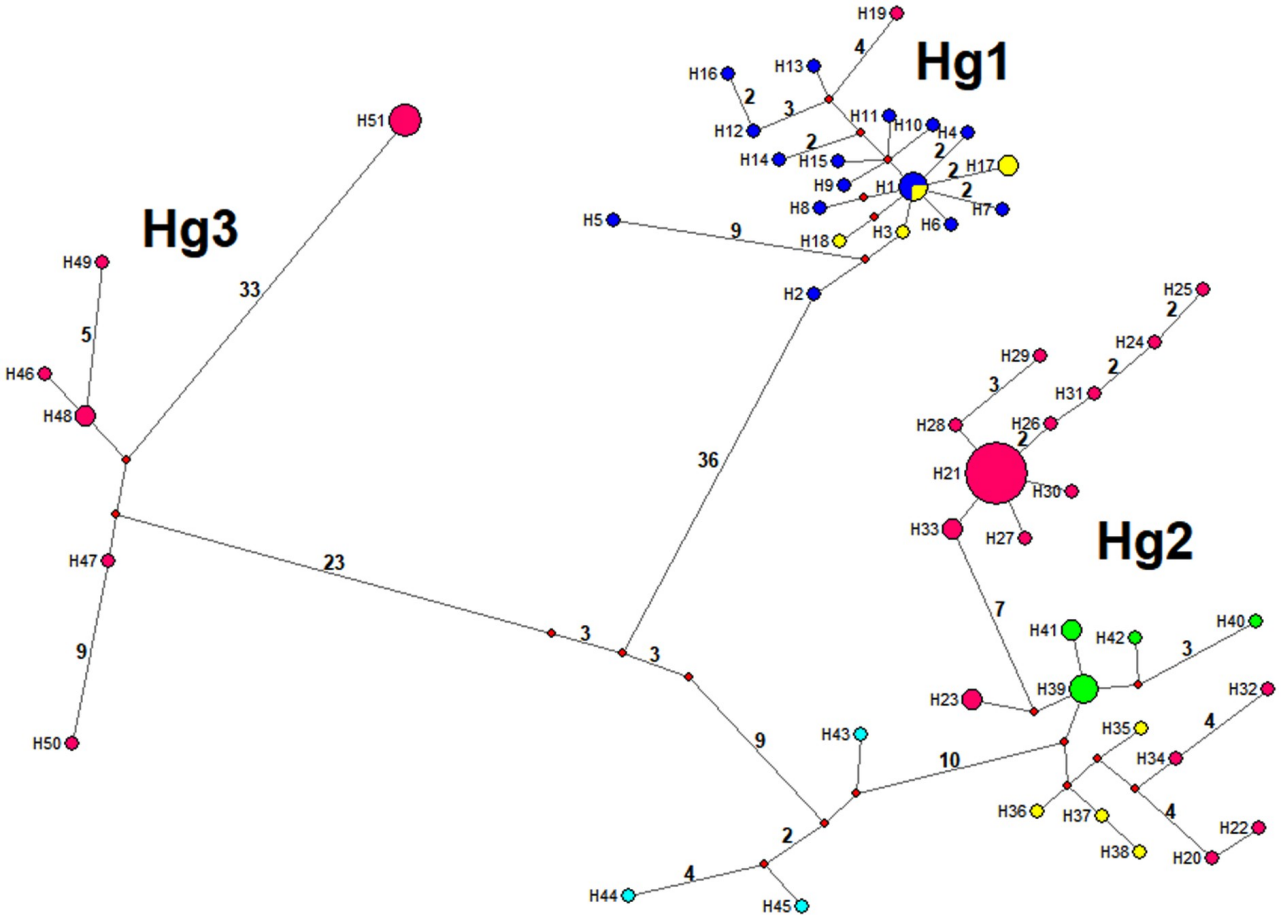
Median-joining haplotype network for the *T*. *dimidiata* complex based on 664 nucleotides of concatenated ND4A + ND4B fragments from Berriozábal + Neotropical Mexican samples. Haplotype frequency is represented by sample size. Haplotype frequency is represented by the size of nodes and missing haplotypes are shown as red circles. The line connecting haplotypes represents one mutational step, whereas numbers along the lines are total number of mutational steps. Colours indicate: blue = Yucatán; yellow = Campeche; green = San Luis Potosí; turquoise = Oaxaca; fuchsia = Chiapas.

The relationship between haplogroups from all Mexican neotropical sites (including Berriozábal) using both concatenated ND4 fragments was analyzed using 51 unique haplotypes (Fig 6). The network distinguishes three haplogroups separated by 48–73 mutational steps. The Hg1 has samples from YP and Palenque (Chiapas), which are separated by at least 50 mutational steps from Hg2 (Playa Vieja, Oaxaca). The Hg3 contains samples from Chiapas, H51 has samples from Berriozábal (Hg3B; Nuevo Progreso and IZ), which are separated from the Pacific coast Chiapas clade (Hg3A; Manacal, Nuevo Horizonte, Tapachula). The greatest number of mutational steps separating haplogroups using the concatenated fragments is between Hg1 and Hg3, not between Hg1 and Hg2, as when only the ND4A is analyzed (including all Central America and Colombia samples).

**Fig 7:**
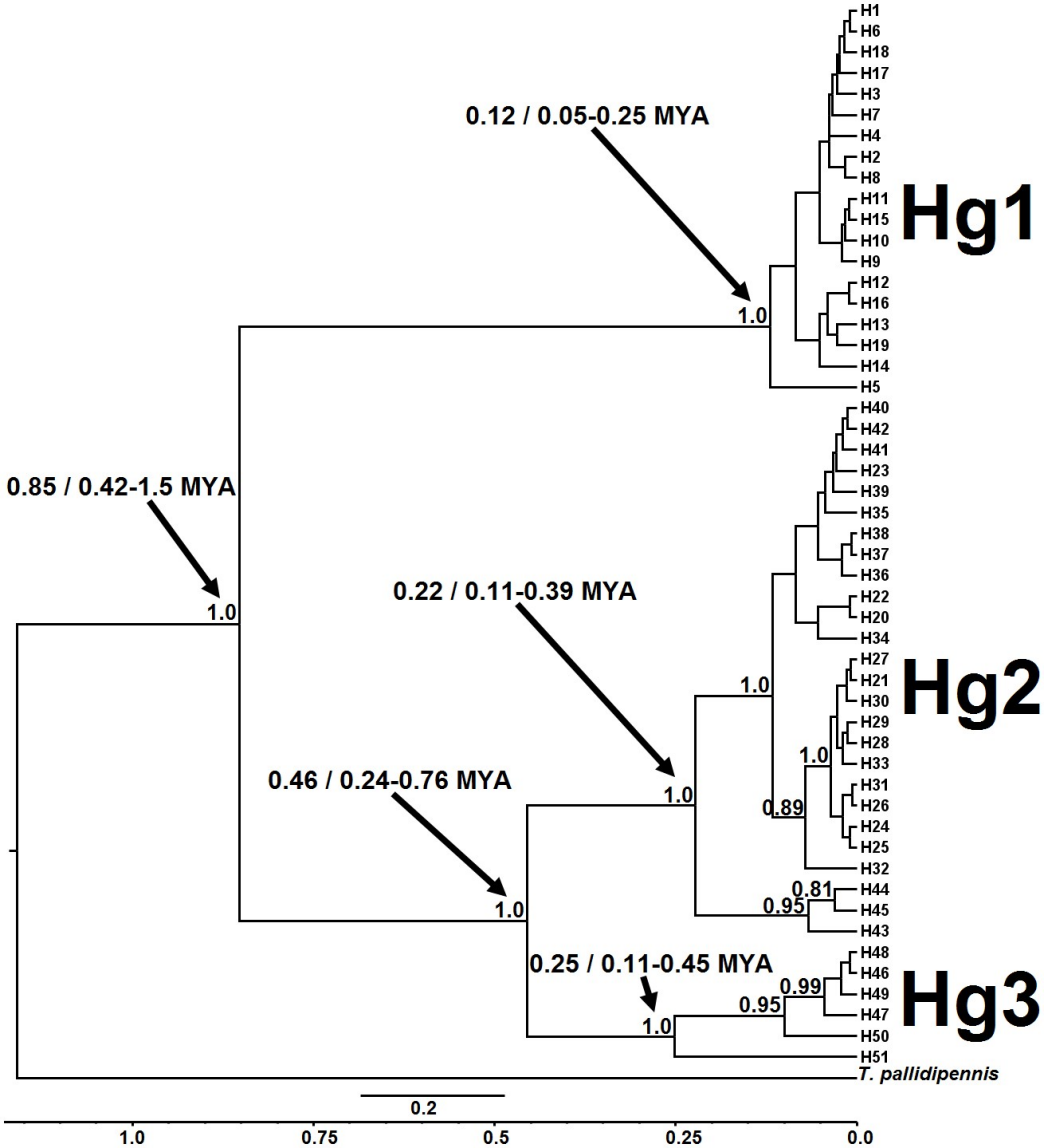
Calibrated maximum-clade-credibility tree for the *T*. *dimidiata* complex using the concatenated ND4A + ND4B fragments from Berriozábal and the Neotropical Mexican region. Numbers above each branch represent PP ≥ 0.95. *T*. *pallidipennis* was used as outgroup. The scale bar represents the expected number of nucleotide substitutions per site and below the line of time in million years ago (MYA) and the numbers indicated by arrows are the time estimate and the 95% HPD.

Divergence time from the concatenated fragments ND4A and ND4B was analyzed using the same 51 unique haplotypes (Fig 7). The most appropriate model was HKY+G (-InL = 2045.9746, Delta BIC = 0, BIC = 5170.6641) with gamma = 0.196. The phylogenetic tree separates again the three primary haplogroups (PP = 1.0), a topology similar to that using only ND4A. The MRCA for the primary divergence was estimated at 0.85 MYA (95% HPD interval = 0.42–1.5 MYA). The combined Hg2-Hg3 clade divergence was estimated at 0.46 MYA (95% HPD interval = 0.24–0.76 MYA), more recent than using the ND4A alone. Secondary divergence of both Hg2 and Hg3 clades occurred prior to that of Hg1, at 0.22 MYA (95% HPD interval = 0.11–0.39 MYA) and 0.25 MYA (95% HPD interval = 0.11–0.45 MYA), respectively. While divergence time of the Hg2 is similar whether analyzed using ND4A alone, or with both fragments, this was not the case for Hg3 (probably due to greater sample bias of only 35 to 60% of Hg3 populations amplifying with the ND4A primers). Hg3 divergence gives rise on the one hand to the current designated clade Hg3B (H51, Berriozábal), and the Hg3A clade, the latter only with haplotypes from the Chiapas Pacific coast. Hg2 divergence gives rise to three distinct sub-clades using the concatenated sequences, the oldest or last to diverge contains Pacific coast populations (Pacific coast west of the Tehuantepec Isthmus), while the primary Hg2 sub-clade further diverges (PP = 1.0) giving rise to one sub-clade containing haplotypes from Berriozábal, and a second sub-clade containing all other Hg2 populations north and west along the Gulf of Mexico coast (San Luis Potosi), northeast (Campeche), and east of the Isthmus (Berriozábal). Divergence of the Hg1 was estimated at 0.12 MYA (95% HPD interval = 0.05–0.25 MYA) using the concatenated fragments.

### Historical population changes of *T*. *dimidiata* haplogroups

**Fig 8:**
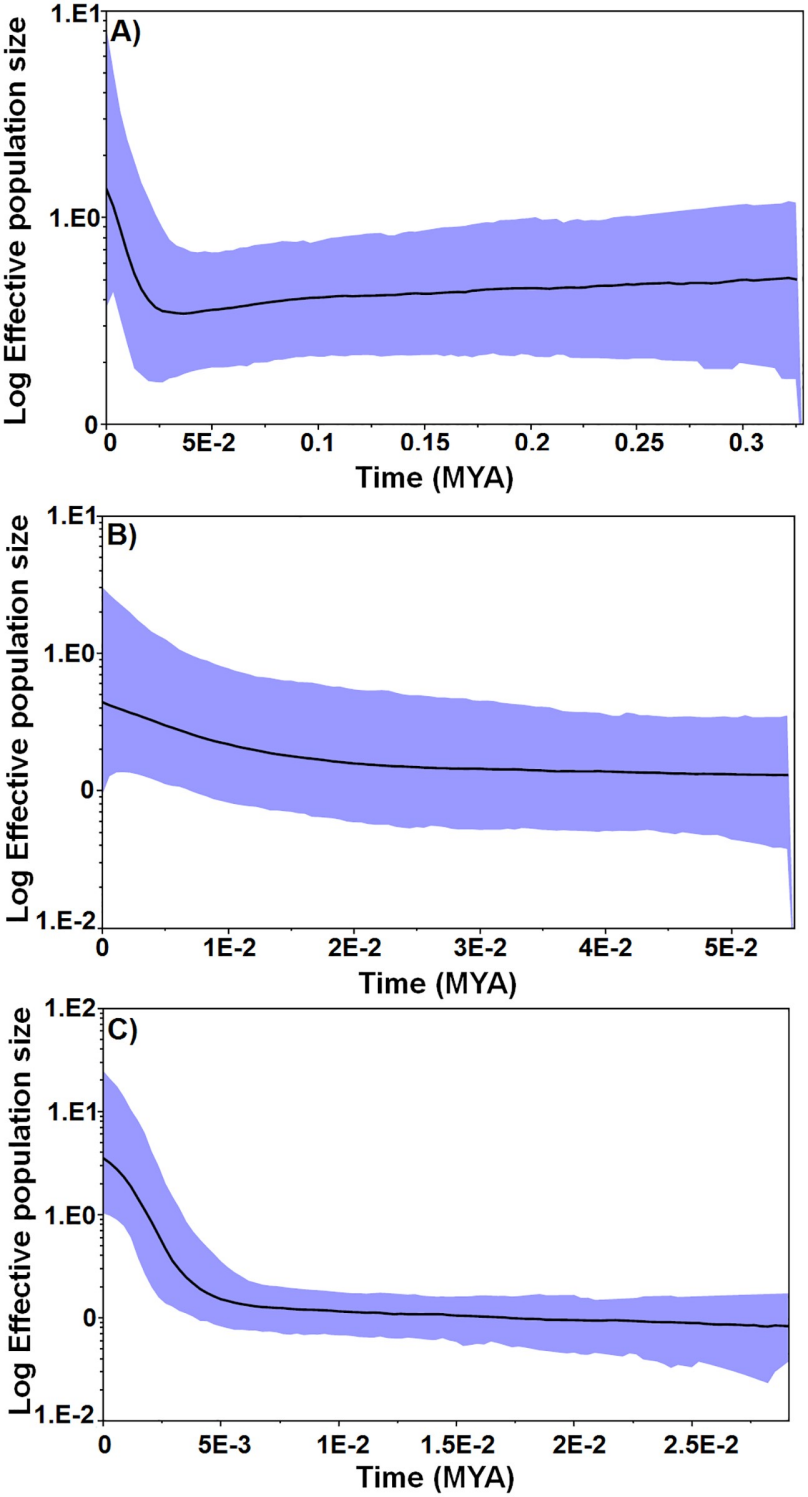
Bayesian skyline plots (BSP) showing the historical demographic changes of *T*. *dimidiata* estimated from the concatenated ND4A + ND4B fragments. The relative population size measured as a product-effective population size (y-axis) is shown over time in millions of years (x-axis) in a simulated coalescent-based demographic model using standard Markov chain Monte Carlo (MCMC). A) the concatenated ND4A + ND4B sequences from Berriozábal and other Mexican Neotropical sites, B) The concatenated ND4A + ND4B sequences Hg2 from Berriozábal and other Mexican Neotropical sites and for C) ND4A from Colombia only Hg3 [19]. The thick black line is the median estimate and the solid (blue) interval shows the 95% highest posterior density limits.

The BSP for the concatenated sequences (ND4A + ND4B) using corrected mutation rates of all haplogroups of only Mexican neotropical populations reported herein, indicates an increase in effective population size approximately 0.025 MYA (Fig 8A). The BSP for Hg2 alone, using the concatenated dataset from the Mexican neotropical sites, also indicates a population increase at approximately 0.02 MYA (Fig 8B). Re-analysis of a Colombian dataset for ND4A alone [19], using a corrected mutation rate, indicates an increase in population size approximately 0.005 MYA (Fig 8C). Since previous studies had suggested much older increases in effective population size, but only used the ND4A fragment, we reanalysed the continental dataset and separately, the CA/Colombian dataset (Hg3) which estimated an MRCA of 0.05 MYA (S2A Fig) and 0.06MYA (S2B Fig), respectively. Since both previous datasets are biased in terms of population representation (reduced representation of Hg2 and Hg3), we hypothesized that the proportional bias in population analysed, would also give a biased estimate of divergence time. An analysis of only ND4A from the present study´s dataset, with no sample bias from original collections, estimates similar effective population increase to that found using the concatenated fragments, 0.03 MYA (S2C Fig).

### *Trypanosoma cruzi* and DTU infection in *T*. *dimidiata* haplogroups

A total of 314 specimens of *T*. *dimidiata* were analyzed for *T*. *cruzi* infection, from 16 communities in Berriozábal. Overall prevalence was 18.8% (Table 4), with significantly more infected in the dry (29.1%) vs. the rainy season (9.6%) (G = 8.801, *df* = 1, *p* ≤ 0.003). *Trypanosoma cruzi* infection in bugs was not associated with higher zoonotic blood sources (animal:human bloodmeal index), although highest human bloodmeal rates (9.5% vs 11.9%) occurred in the region with greatest human population movement (sub-region D; Table 4). Infection prevalence was similar among sub-regions overall, but for Hg2, the only haplogroup collected in all sub-regions, it was not uniform across the communities within sub-regions, with null or lowest rates measured from the southern historically modified landscapes (E) and where there was highest elevation (D) (Table 5). High infection prevalence was found in the most conserved sub-region A, although these differences were not significant (*p* > 0.05). Haplogroup infection frequencies for bugs from Berriozábal or from sub-region A, where all three were sympatric, were similar between Hg1 (33.3%) and Hg2 (31.5%), but no Hg3 infections were recorded from this area (Table 5). In Berriozábal county, infection rates in Hg2 decreased from north to south, coincidental with the modification gradient (Table 5).

**Table 4:** *T*. *cruzi* infection prevalence for the *dimidiata* complex collected in houses from Berriozábal communities, Chiapas.

Sub-Region	Community	N	*T*. *cruzi* infection (%)	Bloodmeal index A:H
	Buenavista	2	100	-
	Ignacio Zaragoza	44	13.6	1.3
	Nuevo Progreso	16	0	-
	Nuevo Chacacal	2	50	-
	Benito Juárez	1	100	-
**A**		**65**	**15.4**	**2.3**
	Nuevo Montecristo	86	40.7	3.2
	Río Blanco	82	1.2	2.8
**B**		**168**	**21.4**	**5.4**
	Benito Quesada	1	0	-
	Nueva Esperanza	1	0	-
	Santa Teresa	10	20	-
	El Sabinito	4	25	-
**C**		**16**	**18.8**	**0**
	Efraín A. Gutierrez	31	12.9	0.8
	Cuchumbac	7	0	0
	Vistahermosa	5	20	-
**D**		**43**	**11.6**	**0.8**
	Berriozábal	13	15.3	-
	San Antonio Bombanó	9	33.3	-
**E**		**22**	**22.7**	**0**
**County**		**314**	**18.8**	**3.8**

**Table 5:** *Trypanosoma cruzi* infection of *T*. *dimidiata* haplogroups Hg1, Hg2, and Hg3 in Berriozábal county by sub-region, in all other Mexican neotropical sites and in the combined Mexican dataset.

Haplogroup	Berriozábal sub-region	*T*. *cruzi* infection % (N)		
		Berriozábal	Other Mexican neotropical	All Mexican neotropical
1		33.3 (3)	90.3% (31)*	85.2% (34)
	A	33.3 (3)	-	-
2		31.5 (70)	43.3% (67)††	38.7% (137)†
	A	41.6 (12)	-	-
	B	35.1 (37)	-	-
	C	16.6 (6)	-	-
	D	14.3 (14)	-	-
	E	0 (1)	-	-
3		0 (13)	77.7% (18)	48.4% (31)
	A	0 (11)	-	-
	B	0 (1)	-	-
	E	0 (1)	-	-
Complex		26.7% (86)	61.2% (116)	48% (202)

**Statistical** X^2^ **significance**:

* = more than expected *p* < 0.001,

^††^ = less than expected *p* < 0.01,

^†^ = less than expected *p* < 0.05.

Comparatively, the infection rate for Hg1 was significantly more than expected in other Mexican neotropical sites, and using the complete dataset (few individuals from Berriozábal county; X^2^ = 11.1, *df* = 2, *p* = 0.0009 and X^2^ = 20.5, *df* = 2, *p* < 0.00001, respectively). In contrast, Hg2 infection prevalence was significantly lower than expected in other Mexican neotropical sites (X^2^ = 9.1, *df* = 2, *p* = 0.003), and in the complete dataset (X^2^ = 4.8, *df* = 2, *p* = 0.03). There was a significant difference in infection prevalence between Berriozábal and the other Mexican neotropical sites for both Hg1 (G = 30.96, *df* = 1, *p* = 2.8 E-8) and Hg2 (G = 18.46, *df* = 1, *p* = 1.7 E-5), while no analysis could be run for Hg3 due to no infection encountered in Berriozábal. There was also a significant difference in infection prevalence in bugs from the complete Mexican neotropical dataset between Hg1 and Hg2 (G = 6.1, *df* = 1, *p* = 0.013), between Hg1 and Hg3 (G = 5.3, *df* = 1, *p* = 0.02), and between Hg2 and Hg3 (G = 22.2, *df* = 1, *p* < 0.0001).

**Fig 9:**
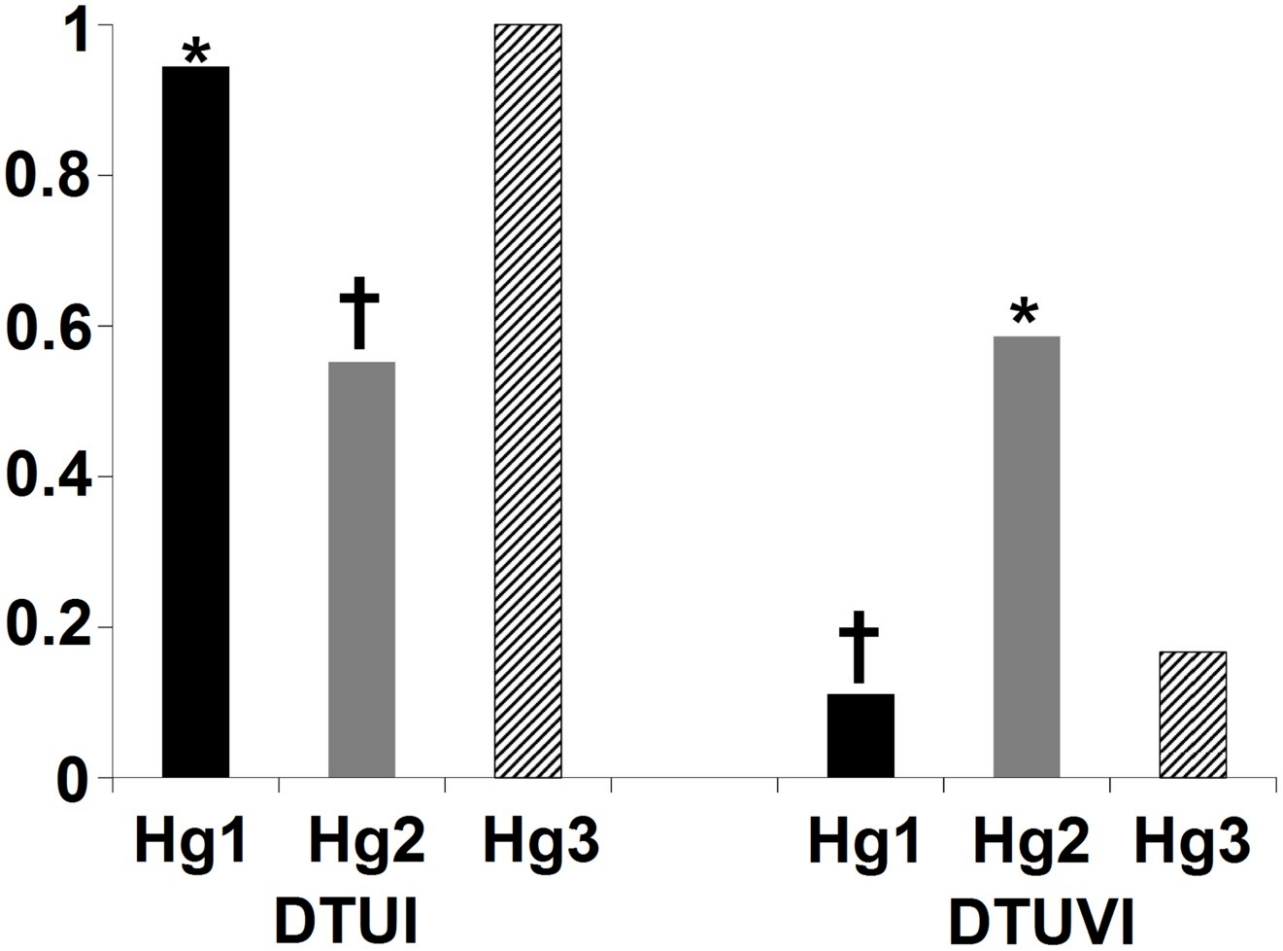
Infection prevalence with DTUI and DTUVI in the three *dimidiata* haplogroups from the Mexican Neotropical region. (X^2^ confidence (95%) for * = more than expected *p* < 0.05, † = less than expected *p* < 0.05).

DTUI and DTUVI frequencies were significantly different among the three *T*. *dimidiata* haplogroups (Fig 9). The DTUI frequency in Hg1 was significantly more than expected compared to the other two haplogroups (X^2^ = 4.029, *df* = 2, *p* = 0.045), while that in Hg2 was significantly less than expected (X^2^ = 5.058, *df* = 2, *p* = 0.025). In contrast, the DTUVI frequency in Hg1 specimens was significantly lower than expected (X^2^ = 5.431, *df* = 2, *p* = 0.02), but more than expected in Hg2 (X^2^ = 5.38, *df* = 2, *p* = 0.02). DTUI infection prevalence was significantly greater to that of DTUVI, in Hg1 (G = 13.2, *df* = 1, *p* = 0.0002) and in Hg3 (G = 3.69, *df* = 1, *p* = 0.05), but not in Hg2, in which the hybrid genotype is amplified equally.

## Discussion

Phylogeographic analyses of Mexican neotropical populations of *T*. *dimidiata* analyzed herein, support previous studies providing evidence for three primary haplogroups (Hg1, Hg2, Hg3) [8, 9, 12, 14, 18, 19, 23, 30, 35]. There is also significant support for further genetic divergence of both Hg2 and Hg3 with clear geographic structuring [18, 35]. A recent re-analysis [36] suggests designation of three species for the *dimidiata* complex which do not coincide with phylogeographic analyses: *T*. *dimidiata* s. s. (includes Hg2 and Hg3 from the present study), *T*. sp. aff. *dimidiata* (Hg1 from the present study) and *T*. sp. aff. *dimidiata*—caves (a cave haplogroup not present in Mexico). This latter proposal does not consider evidence for genetic, phenetic, and ecological niche traits differentiating Hg2 and Hg3 haplogroups. The MRCA for the primary divergence of all current *T*. *dimidiata* haplogroups was dated herein at 0.97 MYA, within the latter half of the middle Pleistocene. This is more recent than another estimate using uncorrected substitution rates (1.1–1.8% per Myr) by Monteiro et al. [18]. The correction is necessary since the loss of transient polymorphisms by selection and drift, particularly for mitochondrial genes, reduces the long-term substitution rate relative to the short-term mutation rate by 10-fold. The first clade to further diverge at 0.60 MYA gave rise to two haplogroups, Hg2 distributed almost exclusively west and north of the Tehuantepec Isthmus (recent collections in the YP are the exception) [5, 8], and Hg3 only distributed east and south of the Tehuantepec Isthmus into northern South America (NSA). Both of these haplogroups further diverged, Hg2 most recently at 0.25 MYA separating Gulf and Pacific coast clades. The second clade, Hg3, had multiple divergence events, the first at 0.44 MYA giving rise to the two currently designated Hg3B (Berriozábal/northern Chiapas, CA and NSA), and Hg3A (Pacific coast Chiapas, Santander and Guajira, Colombia). The former clade, Hg3B, again diverged at 0.25 MYA, around the same time as Hg2, into one sub-clade with populations only from areas around the Chimalapas/Tehuantepec Isthmus (both west and in Berriozábal), and a second sub-clade with all CA and NSA populations. It is important to highlight that Hg3 is the only group successfully dispersing south from southern Mexico to NSA and has the broadest distribution among the three haplogroups [9, 10]. The last major group to diverge was Hg1, the basal clade, at 0.20 MYA, coincidental with other divergence events of all haplogroups, and with the second to last interglacial period. Hg1 distributions are restricted east of the Tehuantepec Isthmus (no collections west to date), predominately into the YP and its continuous northern Guatemala region, but no further south through CA or NSA [8, 9, 14, 36].

Ibarra-Cerdeña et al. [34] estimated that speciation events of Neotropical triatomines occurred principally during the Pleistocene. Indeed, more than nine interglacial cycles of varying magnitude were recorded since 0.97 MYA in the American continent, principally in the NA Nearctic realm, with alternating phases of cold and semi-arid conditions during the glaciations and warm/humid conditions during the interglacial or deglaciation periods [62, 63]. Although less important or existent in the Neotropical Mesoamerican region, northern continental glacial/deglaciation events played an important role on atmospheric conditions in the Neotropics, as well as on landscape fragmentation, mammal extinctions and divergence, provoking population contractions, followed by expansions, and dispersal [64]. Effective population increases of the three majors Mexican haplogroups analyzed together, and separately for Hg2, coincided with the peak of the Last Glacial Maximum (LGM; 0.03–0.02 MYA). This would coincide with the last great faunal extinctions, and subsequent faunal expansions (south and northward), including presence and expansion of humans in the American continent. Mexico was one of the major agricultural crop developers and exporters south to CA and SA during the Holocene [65]. Effective population expansion of Hg3 from Colombian sub-groups is the most recent (0.005 MYA), although this may be preliminary, and should be reanalysed using populations previously analyzed, perhaps using the ND4B fragment to balance regional representation [19].

The driest and coolest period in the Neotropical region (both Mexican coasts and the Yucatan peninsula, south through the Tehuantepec Isthmus/Chimalapas rainforest, to CA) was post-LGM, based on a greater proportion of carbon from tropical forests in the region as compared to LGM, or pre-industrial Holocene [64, 66–68]. The Chimalapas rainforest traverses the Tehuantepec Isthmus, an important biogeographic barrier [69, 70], which is the southern border of the Mexican Trans-volcanic belt and Nearctic realm, and it is the northwest limit of the Mesoamerican biodiversity region, and Neotropical realm. The Mexican neotropical bioregion also extends north only along the Gulf of Mexico through Veracruz, and west along the Pacific coast to Jalisco, but not the intermediate high plains region (between Sierra Madres). All three *T*. *dimidiata* haplogroups have only been collected sympatric (all life stages) in the landscape along the northwest region of Chiapas in Berriozábal, which is the eastern edge of the Chimalapas. This may be a fortuitous collection (in more than one community), or it may be key evidence to help interpret *T*. *dimidiata* complex evolution. Ecological niche models for the three haplogroups overlap in this region [10], and intensive bug collections over representative portions of the Mexican neotropical region over two decades, as well as passive collections reported in other studies over the last 70 yrs, have not reported the three haplogroups sympatric since it was assumed that they would not be reproductively isolated. This leads us to propose that the MRCA of the *T*. *dimidiata* complex evolved in the Chimalapas/Tehuantepec Isthmus region. In order to analyze this hypothesis further, more complete collections of the two minority haplogroups (Hg1 and Hg3) and further genetic analyses of all three haplogroups will be necessary with appropriate sample design for geographic and ecological relevance.

Both at regional (Berriozábal) and Mexican neotropical geographic scales, all haplogroups were significantly differentiated (F_ST_ > 0.7), indicating an insignificant amount of genetic exchange among them. Similar differentiation has been reported previously using ND4A and nuclear markers [4, 9, 13, 18, 19]. However, greatest differentiation was estimated herein between Hg1 and Hg3 using both fragments, in contrast to average but highest differentiation between Hg1 and Hg2 reported in a previous study, albeit with a sample representation bias [14], and herein using only the ND4A. Hence, care should be taken to interpret data solely using this latter fragment alone. The evidence further supports the clear differentiation of Hg2 and Hg3 for future systematic revision and species designations, which should also consider the vast evidence for phenetic distinctions, most recently using evidence for *dimidiata* haplogroup interactions mediated by volatile compounds [30–32]. Antennal phenotype was significantly different between Hg2 and Hg3 females, and significantly different between Hg1 and Hg3 males [32], whileHg3 (specifically Hg3A) released significantly fewer alarm compounds as compared to both Hg1 and Hg2 [30].

### Genetic diversity of haplogroups

At a regional analytical scale (Berriozábal county), lowest genetic diversity (haplotypic and nucleotide), with evidence of bottlenecks, occurs in the most anthropically modified and transited eastern and southern sub-regions. Highest diversity for Hg2 (the most abundant haplogroup in Berriozábal county) was, as expected, in the most environmentally conserved sub-regions, with moderate to high haplotype, although low nucleotide diversity, indicating at least specifically for this haplogroup, recent bottlenecks [71]. This trend was also evident in other regions where it is distributed in Mexico [5]. Hg2, the second most widely distributed haplogroup of the complex, is distributed along two principal axes only in Mexico naturally (1) the Gulf of Mexico coast and northern foothills of the Eastern Sierra Madre, and (2) the Pacific coast and foothills west and south of the Western Sierra Madre from Colima to the Isthmus in Oaxaca. Both of these distributions are west and north of the Tehuantepec Isthmus. Recent Hg2 dispersal to the YP may have occurred due to human migratory trends to and from the Chol and Tzeltal populations in Tabasco and northern Chiapas (northern Tehuantepec Isthmus) to the YP and from human migrations in the last 50 yrs, an important consideration for vector control programs [5, 8, 41]. Hg1 is the oldest of the lineages, distributed in the YP, northern Chiapas (Palenque National Park and Arqueological area only, northern Berriozábal) and northern Guatemala, and has high genetic diversity and expanding populations. This basal haplogroup has the second narrowest distribution of the complex, *T*. *hegneri* having the most restricted [10].

Diversity of the Hg3 from the Pacific coast of Chiapas (Hg3A) was higher than that of Hg3B from Berriozábal (only one haplotype). Hg3 is distributed east and south of the Tehuantepec Isthmus through CA to Colombia and other NSA countries. It has also been reported from the northern edge of the Tehuantepec Isthmus in Tuxpan, Veracruz [8], where the Grijalva river (origin in Huehuetenango, Guatemala), opens into the Gulf of Mexico. The Grijalva runs west and north from Guatemala through the northern Chiapas mountain range and borders Berriozábal´s sub-region A. Its course is a common human migratory route, also used to transport wood, exotic species, agricultural products, and commerce [72, 73]. Hg3 diverged approximately 0.44 MYA into two subgroups, the first designated Hg3B distributed from the Chimalapas/Grijalva river basin of northern Chiapas, through CA, to Colombia, Peru, and Ecuador. The Hg3A is only found in one restricted region of the southern Pacific Soconusco coast of Chiapas in Mexico (no more than a few samples collected across the Suchiate river in San Marcos in the Mexico-Guatemala border region) and in two Colombian regions (Guajira and Santander) [9]. More importantly, it is absent from all other regions in Chiapas where the Hg3B occurs, and any region thus far analyzed in the Mexican Neotropics. Phylogenetic inferences could be explained based on the historical population exchange that has occurred between this pre-classical Mayan region (Izapa, Chiapas) with Incan populations of Colombia, and may have established either in Chiapas or Colombia through cultural/agricultural hitch-hiking and a founder effect. Since the Hg3 is the only haplogroup shared from Mexico to NSA, information regarding its population structure, dispersal ecology, and vector capacity will be important to design, monitor, and analyze regional vector control and *T*. *cruzi* transmission prevention.

### Vector capacity and DTU specificity in *dimidiata* haplogroups

Hg1 and Hg2 infection frequencies for bugs from Berriozábal and from sub-region A (where the three are sympatric in two modified landscapes) were similar, both significantly higher than Hg3. Hg1 infection was also significantly more prevalent than expected in the rest of the Mexican neotropical region (double that of Hg2), while Hg2 infection was significantly less than expected overall, but similar to that in Berriozábal. All pairwise differences in haplogroup infections were significant using the complete Mexican dataset. Since there is important mammal species turnover where the three haplogroups are distributed, differences in *T*. *cruzi* infection may not be related to intrinsic specificity, but may be due to differing infection rates in reservoirs. However, data from synanthropic rodents present in all three distribution regions indicate similar infection prevalence across the region which does not coincide with haplogroup infection differences based on host infections. There was also similar infection prevalence in bats in areas with either Hg1 or Hg2 [5, 6, 74].

*Trypanosoma cruzi* infection in bugs overall was not associated with higher zoonotic blood sources, although in the domestic habitat, there was high mixed human-zoonotic bloodmeal rates. The availability and mix of bloodmeal sources may provide the opportunity for vector population amplification, and a benefit for *T*. *cruzi* maintenance in human modified habitats. *Triatoma dimidiata* (Hg3) collected from houses in Guatemala, (examined using ELISA), had fed most on humans, although mixed meals with opossums and cows were also reported [75], while studies of feeding patterns have frequently documented multiple hosts by domestic insects [76–79]. Bugs from Berriozábal having only human or mixed bloodmeals, had predominantly *T*. *cruzi* DTUI, while those with only zoonotic sources had both DTUI and DTUVI [80]. In addition to feeding patterns, insect density and spatial and temporal occurrence of hosts and insects contribute to host choice [81], and the number, identity, and proximity of synanthropic species also influence feeding patterns [77]. Biotic interaction models for all haplogroups predict significant links are strongest with synanthropic mammal reservoirs [39], an important indicator for *T*. *cruzi* transmission exposure hazard [41]. The threshold for reservoir diversity and abundance below which *T*. *cruzi* or specific DTUs are no longer transmitted, has yet to be analyzed.

Independent of infection prevalence, a triatomine´s capacity to sustain one DTU, may not be similar to another, but may be also related to the presence or efficacy of parasite acquisition from reservoir communities. DTUI and DTUVI frequencies were significantly different among the three *T*. *dimidiata* haplogroups. DTUI and DTUVI infection rates in Hg1 and Hg3 were significantly different, while there was no difference between them in Hg2, indicating DTUVI amplification in this latter haplogroup. The DTUI frequency in Hg1 was significantly more than expected, while that in Hg2 was significantly less. In contrast, DTUVI frequency in Hg1 specimens was significantly less, while more than expected in Hg2. Not only is the prevalence of *T*. *cruzi* DTUs among haplogroups different, but specific parasite haplotypes may be haplogroup-specific. A unique DTUI haplotype found only in Hg2 in IZ/sub-region A, was not found in any of the few Hg1 and Hg3 infected specimens from the same site. Additionally, two unique haplotypes of DTUVI were identified in Hg2 from Berriozábal, and only from one other site in the Mexican Neotropics, on the western edge of the Chimalapas/Tehuantepec Isthmus; the zoonotic source of these haplotypes has yet to be identified [74, 80]. There was no genetic differentiation of DTUI (both 18S and ME haplotypes) from sylvatic or human reservoirs, and bugs, within Berriozábal, although there was genetic differentiation of these populations with those from Palenque (eastern Chiapas, Hg2 and Hg1), from Tapachula (southern Pacific coast Chiapas, Hg3A), and from southern Campeche (Hg1 and Hg2) [74, 80]. This information is important for vector control strategies, since not only are there specific vector populations expanding along the socioeconomic gradient, but they are transmitting distinct parasite populations, despite relative proximity (300–1000 km).

There is sufficient genetic evidence currently, not only to distinguish the three principal *T*. *dimidiata* haplogroups, but also to identify evolving differences in parasite populations, a key component of vector capacity. Characterizing and understanding bug haplogroups (genetic and phenetic traits), and this species complex´ evolution, will allow us to analyze associations of life history traits with population amplification across regions, and expansion potential across each haplogroup´s distribution. It also allows us to understand the impact of human-modification and practices within landscapes, and the transmission of specific parasite populations to the human population, as well as other synanthropic reservoirs.

On a final note, preliminary studies regarding the *dimidiata* complex population genetics in Mexico, alerted us to a bias in annealing of parasite DNA to the existing ND4 primers, which were designed based on a large fragment of the ND4 gene [42]. This bias occurred differentially depending on the collection site in Mexico, which also coincided with haplogroup type. This bias is probably based on an increase in nucleotide polymorphisms at primer binding sites of the originally designed ND4 oligonucleotides (herein ND4A), and therefore in order to reduce bias for landscape genetic studies, and phylogeography, a complementary fragment of the same gene was identified and used. Although principal phylogenetic topology is the same using only the previously existing fragment (ND4A), the use of the two fragments (concatenated ND4A and ND4B), reveals greater structure of more recent divergence of sub-clades (i.e. 3 within Hg2 and potentially 3 in Hg3). Together, both fragments eliminate the bias regarding proportional sample representation across geographic sites, and provide more precise analysis of genetic diversity and differentiation particularly at the landscape scale, since the ND4B is more polymorphic. Additionally, there is an important bias in network analyses using the ND4A alone, since Hg1 is the most distant from Hg3, when the concatenated fragments are used. Effective population increases, probably biased by haplogroup representation in sample sets, was double when the ND4A alone was used. Hence, recent regional and landscape level population shifts cannot be analyzed appropriately using the ND4A alone. Other mitochondrial markers, also used in previous studies (cyt *b*, COI) may have similar bias.

*Trypanosoma cruzi* infection prevalence in the Berriozábal region was associated with a reduction from north to south, which also coincided with conserved to highly modified landscapes, and highest human bloodmeal rates (occurring in the region with greatest human population movement). Understanding gene flow and migration of insect vectors is critical to effective control and elimination of vector-borne diseases, and in the present study we have contributed to a growing body of evidence regarding differences among the three *dimidiata* complex haplogroups. These primary haplogroups have different traits related to ecological niche and distribution, persistence or expansion in modified habitats, modification of food source abundance in anthropic habitats, and *T*. *cruzi* infection and DTU specificity. At least Hg1 has expanding populations in Mexico, an alarm bell for vector control programs. Despite the presence of the *dimidiata* complex across the Neotropical region of Mexico, the three main haplogroups are sympatric only in the western region of Chiapas, which is the eastern fringe of the Chimalapas rainforest, primary ecotope of the Tehuantepec Isthmus. The Isthmus is an important biogeographic barrier diverging many taxa west and east between 2.4 and 10 MYA [69, 70]. In the present study, there is evidence of a significant difference of *T*. *cruzi* lineages in the three *T*. *dimidiata* haplogroups. Knowledge of the parasite´s genetic diversity in an area is important to analyze efficacy of therapeutic tools, since *T*. *cruzi* DTU or haplotype may be associated with cardiomyopathy manifestations, parasitemia in infected individuals, or susceptibility to pharmacological agents [82]. Vector diversity has direct implications for the design and success of vector control strategies of the dimidiata complex in Mexico and the Mesoamerican biodiversity region [83].

## Supporting information

S1 Table Origin of *Triatoma dimidiata* samples included in analyses. (XLSX)

S1 Fig Summary statistics for *dimidiata* haplogroups from Berriozábal sub-regions, using ND4A and ND4B fragments. (TIF)

S2 Fig Bayesian skyline plots (BSP) showing historical demographic changes of *T*. *dimidiata* estimated from the ND4A fragment. The relative population size measured as a product-effective population size (y-axis) is shown over time in millions of years (x-axis) in a simulated coalescent-based demographic model using standard Markov Chain Monte Carlo (MCMC). A) ND4A from Berriozábal + other Mexican Neotropical sites + continental GenBank sequences, B) ND4A from CA and Colombia only Hg3, C) ND4A from Berriozábal + other Mexican Neotropical sites. The thick black line is the median estimate and the solid (blue) interval shows the 95% highest posterior density limits.
(TIF)

S1 File Haplotypes of the ND4B fragment. (DOCX)

## References

[1] Abad-Franch F, Paucar A, Carpio C, Cuba CA, Aguilar HM, Miles MA. Biogeography of Triatominae (Hemiptera: Reduviidae) in Ecuador: implications for the design of control strategies. Memorias do Instituto Oswaldo Cruz. 2001; 96(5):611–20. 11500757 10.1590/s0074-02762001000500004

[2] Monroy MC, Bustamante DM, Rodas AG, Enriquez ME, Rosales RG. Habitats, dispersion and invasion of sylvatic *Triatoma dimidiata* (Hemiptera: Reduviidae: Triatominae) in Peten, Guatemala. Journal of Medical Entomology. 2003; 40(6):800–6. 14765656 10.1603/0022-2585-40.6.800

[3] Ibarra-Cerdena CN, Sanchez-Cordero V, Townsend Peterson A, Ramsey JM. Ecology of North American Triatominae. Acta Tropica. 2009; 110(2–3):178–86. 10.1016/j.actatropica.2008.11.012 19084490

[4] Grisales N, Triana O, Angulo V, Jaramillo N, Parra-Henao G, Panzera F, et al Diferenciación genética de tres poblaciones colombianas de *Triatoma dimidiata* (Latreille, 1811) mediante análisis molecular del gen mitocondrial ND4. Biomedica. 2010; 30:207–14. 20890568

[5] Lopez-Cancino SA, Tun-Ku E, De la Cruz-Felix HK, Ibarra-Cerdena CN, Izeta-Alberdi A, Pech-May A, et al Landscape ecology of *Trypanosoma cruzi* in the southern Yucatan Peninsula. Acta Tropica. 2015; 151:58–72. 10.1016/j.actatropica.2015.07.021 26219998

[6] Izeta-Alberdi A, Ibarra-Cerdena CN, Moo-Llanes DA, Ramsey JM. Geographical, landscape and host associations of *Trypanosoma cruzi* DTUs and lineages. Parasites & Vectors. 2016; 9(1):631. 27923409 10.1186/s13071-016-1918-2 PMC5142175

[7] Guhl F. Enfermedad de Chagas: Realidad y perspectivas. Biomedica:. 2009; 20:228–34.

[8] Dorn PL, Calderon C, Melgar S, Moguel B, Solorzano E, Dumonteil E, et al Two distinct *Triatoma dimidiata* (Latreille, 1811) taxa are found in sympatry in Guatemala and Mexico. PLoS Neglected Tropical Diseases. 2009; 3(3):e393 10.1371/journal.pntd.0000393 19274073 PMC2648038

[9] Gomez-Palacio A, Arboleda S, Dumonteil E, Townsend Peterson A. Ecological niche and geographic distribution of the Chagas disease vector, *Triatoma dimidiata* (Reduviidae: Triatominae): Evidence for niche differentiation among cryptic species. Infection, Genetics and Evolution. 2015; 36:15–22. 10.1016/j.meegid.2015.08.035 26321302

[10] Ramsey JM, Peterson AT, Carmona-Castro O, Moo-Llanes DA, Nakazawa Y, Butrick M, et al Atlas of Mexican Triatominae (Reduviidae: Hemiptera) and vector transmission of Chagas disease. Memorias do Instituto Oswaldo Cruz. 2015; 110(3):339–52. 10.1590/0074-02760140404 25993505 PMC4489471

[11] Bargues MD, Marcilla A, Ramsey JM, Dujardin JP, Schofield CJ, Mas-Coma S. Nuclear rDNA-based molecular clock of the evolution of triatominae (Hemiptera: reduviidae), vectors of Chagas disease. Memorias do Instituto Oswaldo Cruz. 2000; 95(4):567–73. 10904416 10.1590/s0074-02762000000400020

[12] Marcilla A, Bargues MD, Ramsey JM, Magallon-Gastelum E, Salazar-Schettino PM, Abad-Franch F, et al The ITS-2 of the nuclear rDNA as a molecular marker for populations, species, and phylogenetic relationships in Triatominae (Hemiptera: Reduviidae), vectors of Chagas disease. Molecular Phylogenetics and Evolution. 2001; 18(1):136–42. 10.1006/mpev.2000.0864 11161750

[13] Harris K. Taxonomy and Phylogeny of North American Triatominae: Public Health Implications. Atlanta (GA): Moorehouse School of Medicine; 2003.

[14] Bargues MD, Klisiowicz DR, Gonzalez-Candelas F, Ramsey JM, Monroy C, Ponce C, et al Phylogeography and genetic variation of *Triatoma dimidiata*, the main Chagas disease vector in Central America, and its position within the genus *Triatoma*. PLoS Neglected Tropical Diseases. 2008; 2(5):e233 10.1371/journal.pntd.0000233 18461141 PMC2330091

[15] Tamay-Segovia P, Alejandre-Aguilar R, Martinez F, Villalobos G, de la Serna FJ, de la Torre P, et al Two *Triatoma dimidiata* clades (Chagas disease vector) associated with different habitats in southern Mexico and Central America. The American Journal of Tropical Medicine and Hygiene. 2008; 78(3):472–8. 18337346

[16] Herrera-Aguilar M, Be-Barragan LA, Ramirez-Sierra MJ, Tripet F, Dorn P, Dumonteil E. Identification of a large hybrid zone between sympatric sibling species of *Triatoma dimidiata* in the Yucatan Peninsula, Mexico, and its epidemiological importance. Infection, Genetics and Evolution. 2009; 9(6):1345–51. 10.1016/j.meegid.2009.09.009 19786121

[17] Blandon-Naranjo M, Zuriaga MA, Azofeifa G, Zeledon R, Bargues MD. Molecular evidence of intraspecific variability in different habitat-related populations of *Triatoma dimidiata* (Hemiptera: Reduviidae) from Costa Rica. Parasitology Research. 2010; 106(4):895–905. 10.1007/s00436-010-1762-9 20165880

[18] Monteiro FA, Peretolchina T, Lazoski C, Harris K, Dotson EM, Abad-Franch F, et al Phylogeographic pattern and extensive mitochondrial DNA divergence disclose a species complex within the Chagas disease vector *Triatoma dimidiata*. PloS one. 2013; 8(8):e70974 10.1371/journal.pone.0070974 23940678 PMC3733668

[19] Gomez-Palacio A, Triana O. Molecular evidence of demographic expansion of the chagas disease vector *Triatoma dimidiata* (Hemiptera, Reduviidae, Triatominae) in Colombia. PLoS Neglected Tropical Diseases. 2014; 8(3):e2734 10.1371/journal.pntd.0002734 24625572 PMC3953067

[20] Dorn PL, de la Rua NM, Axen H, Smith N, Richards BR, Charabati J, et al Hypothesis testing clarifies the systematics of the main Central American Chagas disease vector, *Triatoma dimidiata* (Latreille, 1811), across its geographic range. Infection, Genetics and Evolution. 2016; 44:431–43. 10.1016/j.meegid.2016.07.046 27496718 PMC5025387

[21] Justi SA, Galvao C. The Evolutionary Origin of Diversity in Chagas Disease Vectors. Trends in Parasitology. 2017; 33(1):42–52. 10.1016/j.pt.2016.11.002 27986547 PMC5518462

[22] Bustamante DM, Monroy C, Menes M, Rodas A, Salazar-Schettino PM, Rojas G, et al Metric variation among geographic populations of the Chagas vector *Triatoma dimidiata* (Hemiptera: Reduviidae: Triatominae) and related species. Journal of Medical Entomology. 2004; 41(3):296–301. 15185928 10.1603/0022-2585-41.3.296

[23] Lehmann P, Ordonez R, Ojeda-Baranda R, de Lira JM, Hidalgo-Sosa L, Monroy C, et al Morphometric analysis of *Triatoma dimidiata* populations (Reduviidae:Triatominae) from Mexico and Northern Guatemala. Memorias do Instituto Oswaldo Cruz. 2005; 100(5):477–82. 16184224 10.1590/s0074-02762005000500006

[24] Dorn PL, Monroy C, Curtis A. *Triatoma dimidiata* (Latreille, 1811): a review of its diversity across its geographic range and the relationship among populations. Infection, Genetics and Evolution. 2007; 7(2):343–52. 10.1016/j.meegid.2006.10.001 17097928

[25] Zeledon R. Triatoma dimidiata (Latreille, 1811) y su relación con la enfermedad de Chagas. San José, Costa Rica: Universidad Estatal a Distancia (EUNED); 1981.

[26] Calderon Fernandez G, Juarez MP, Ramsey J, Salazar Schettino PM, Monroy MC, Ordonez R, et al Cuticular hydrocarbon variability among *Triatoma dimidiata* (Hemiptera: Reduviidae) populations from Mexico and Guatemala. Journal of Medical Entomology. 2005; 42(5):780–8. 16363161

[27] Fernandez GC, Juarez MP, Monroy MC, Menes M, Bustamante DM, Mijailovsky S. Intraspecific variability in *Triatoma dimidiata* (Hemiptera: Reduviidae) populations from Guatemala based on chemical and morphometric analyses. Journal of Medical Entomology. 2005; 42(1):29–35. 15691005 10.1093/jmedent/42.1.29

[28] Panzera F, Ferrandis I, Ramsey J, Ordonez R, Salazar-Schettino PM, Cabrera M, et al Chromosomal variation and genome size support existence of cryptic species of *Triatoma dimidiata* with different epidemiological importance as Chagas disease vectors. Tropical Medicine & International Health. 2006; 11(7):1092–103. 16827710 10.1111/j.1365-3156.2006.01656.x

[29] Panzera F, Perez R, Panzera Y, Ferrandis I, Ferreiro MJ, Calleros L. Cytogenetics and genome evolution in the subfamily Triatominae (Hemiptera, Reduviidae). Cytogenetic and Genome Research. 2010; 128(1–3):77–87. 10.1159/000298824 20407223

[30] May-Concha I, Rojas JC, Cruz-Lopez L, Ibarra-Cerdena CN, Ramsey JM. Volatile compound diversity and conserved alarm behaviour in *Triatoma dimidiata*. Parasites & Vectors. 2015; 8:84. 25656170 10.1186/s13071-015-0678-8 PMC4324405

[31] May-Concha I, Rojas JC, Cruz-Lopez L, Millar JG, Ramsey JM. Volatile compounds emitted by *Triatoma dimidiata*, a vector of Chagas disease: chemical analysis and behavioural evaluation. Medical and Veterinary Entomology. 2013; 27(2):165–74. 10.1111/j.1365-2915.2012.01056.x 23205718

[32] May-Concha I, Guerenstein PG, Ramsey JM, Rojas JC, Catala S. Antennal phenotype of Mexican haplogroups of the *Triatoma dimidiata* complex, vectors of Chagas disease. Infection, Genetics and Evolution. 2016; 40:73–9. 10.1016/j.meegid.2016.02.027 26921798

[33] Garcia M, Menes M, Dorn PL, Monroy C, Richards B, Panzera F, et al Reproductive isolation revealed in preliminary crossbreeding experiments using field collected *Triatoma dimidiata* (Hemiptera: Reduviidae) from three ITS-2 defined groups. Acta Tropica. 2013; 128(3):714–8. 10.1016/j.actatropica.2013.09.003 24041592 PMC3840729

[34] Ibarra-Cerdena CN, Zaldivar-Riveron A, Peterson AT, Sanchez-Cordero V, Ramsey JM. Phylogeny and niche conservatism in North and Central American triatomine bugs (Hemiptera: Reduviidae: Triatominae), vectors of Chagas’ disease. PLoS Neglected Tropical Diseases. 2014; 8(10):e3266 10.1371/journal.pntd.0003266 25356550 PMC4214621

[35] Monteiro FA, Weirauch C, Felix M, Lazoski C, Abad-Franch F. Evolution, Systematics, and Biogeography of the Triatominae, Vectors of Chagas Disease. Advances in Parasitology. 2018; 99:265–344. Epub 2018/03/14. 10.1016/bs.apar.2017.12.002 29530308

[36] Justi SA, Cahan S, Stevens L, Monroy C, Lima-Cordon R, Dorn PL. Vectors of diversity: Genome wide diversity across the geographic range of the Chagas disease vector *Triatoma dimidiata* sensu lato (Hemiptera: Reduviidae). Molecular Phylogenetics and Evolution. 2018; 120:144–50. 10.1016/j.ympev.2017.12.016 29248626 PMC5991476

[37] Hotez PJ, Dumonteil E, Woc-Colburn L, Serpa JA, Bezek S, Edwards MS, et al Chagas disease: "the new HIV/AIDS of the Americas". PLoS Neglected Tropical Diseases. 2012; 6(5):e1498 10.1371/journal.pntd.0001498 22666504 PMC3362306

[38] Ramsey JM, Elizondo-Cano M, Sanchez-Gonzalez G, Pena-Nieves A, Figueroa-Lara A. Opportunity cost for early treatment of Chagas disease in Mexico. PLoS Neglected Tropical Diseases. 2014; 8(4):e2776 10.1371/journal.pntd.0002776 24743112 PMC3990484

[39] Sanchez-Gonzalez G, Figueroa-Lara A, Elizondo-Cano M, Wilson L, Novelo-Garza B, Valiente-Banuet L, et al Cost-Effectiveness of Blood Donation Screening for *Trypanosoma cruzi* in Mexico. PLoS Neglected Tropical Diseases. 2016; 10(3):e0004528 10.1371/journal.pntd.0004528 27002523 PMC4803194

[40] Ibarra-Cerdena CN, Valiente-Banuet L, Sanchez-Cordero V, Stephens CR, Ramsey JM. *Trypanosoma cruzi* reservoir-triatomine vector co-occurrence networks reveal meta-community effects by synanthropic mammals on geographic dispersal. PeerJ. 2017; 5:e3152 10.7717/peerj.3152 28413725 PMC5391790

[41] Valdez-Tah A, Huicochea-Gómez L, Nazar-Beatelspacher DA, Ortega-Canto J, Ramsey JM. Human vulnerability for *Trypanosoma cruzi* vector transmission in health and disease processes and social territorial appropriation framework. Salud Colectiva. 2015; 11:191–210. 26172096 10.18294/sc.2015.683

[42] Dotson EM, Beard CB. Sequence and organization of the mitochondrial genome of the Chagas disease vector, *Triatoma dimidiata*. Insect Molecular Biology. 2001; 10(3):205–15. 11437912 10.1046/j.1365-2583.2001.00258.x

[43] Lent H, Wygodzinsky P. Revision of the Triatominae (Hemiptera, Reduviidae), and their significance as vectors of Chagas’ disease. Bulletin of the American Museum of Natural History. 1979; 163:123–520.

[44] Ramsey JM, Gutierrez-Cabrera AE, Salgado-Ramirez L, Peterson AT, Sanchez-Cordero V, Ibarra-Cerdena CN. Ecological connectivity of *Trypanosoma cruzi* reservoirs and *Triatoma pallidipennis* hosts in an anthropogenic landscape with endemic Chagas disease. PloS one. 2012; 7(9):e46013 Epub 2012/10/11. 10.1371/journal.pone.0046013 23049923 PMC3458814

[45] Danielson BJ. Communities in a Landscape: The Influence of Habitat Heterogeneity on the Interactions between Species. The American Naturalist. 1991; 138(5):1105–20.

[46] Botero LA, Mejia AM, Triana O. [Biological and genetic characterization of two Colombian clones of *Trypanosoma cruzi* groups I and II]. Biomedica. 2007; 27 Suppl 1:64–74. Caracterizacion biologica y genetica de dos clones pertenecientes a los grupos I y II de Trypanosoma cruzi de Colombia. 18154246

[47] Noyes HA, Stevens JR, Teixeira M, Phelan J, Holz P. A nested PCR for the ssrRNA gene detects *Trypanosoma binneyi* in the platypus and *Trypanosoma* sp. in wombats and kangaroos in Australia. International Journal for Parasitology. 1999; 29(2):331–9. 10221634 10.1016/s0020-7519(98)00167-2

[48] Da Silva FM, Noyes H, Campaner M, Junqueira AC, Coura JR, Anez N, et al Phylogeny, taxonomy and grouping of *Trypanosoma rangeli* isolates from man, triatomines and sylvatic mammals from widespread geographical origin based on SSU and ITS ribosomal sequences. Parasitology. 2004; 129(Pt 5):549–61. 15552400 10.1017/s0031182004005931

[49] Sokal RR, Rohlf FJ. Biometry: The Principles and Practice of Statistics in Biological Research. New York, NY 1995.

[50] Kumar S, Stecher G, Tamura K. MEGA7: Molecular Evolutionary Genetics Analysis Version 7.0 for Bigger Datasets. Molecular biology and evolution. 2016; 33(7):1870–4. 10.1093/molbev/msw054 27004904 PMC8210823

[51] Librado P, Rozas J. DnaSP v5: a software for comprehensive analysis of DNA polymorphism data. Bioinformatics. 2009; 25(11):1451–2. 10.1093/bioinformatics/btp187 19346325

[52] Fu YX. New statistical tests of neutrality for DNA samples from a population. Genetics. 1996; 143(1):557–70. 8722804 10.1093/genetics/143.1.557 PMC1207287

[53] Tajima F. Statistical method for testing the neutral mutation hypothesis by DNA polymorphism. Genetics. 1989; 123(3):585–95. 2513255 10.1093/genetics/123.3.585 PMC1203831

[54] Excoffier L, Lischer HE. Arlequin suite ver 3.5: a new series of programs to perform population genetics analyses under Linux and Windows. Molecular Ecology Resources. 2010; 10(3):564–7. 10.1111/j.1755-0998.2010.02847.x 21565059

[55] Bandelt HJ, Forster P, Rohl A. Median-joining networks for inferring intraspecific phylogenies. Molecular Biology and Evolution. 1999; 16(1):37–48. 10.1093/oxfordjournals.molbev.a026036 10331250

[56] Drummond AJ, Suchard MA, Xie D, Rambaut A. Bayesian phylogenetics with BEAUti and the BEAST 1.7. Molecular Biology and Evolution. 2012; 29(8):1969–73. 10.1093/molbev/mss075 22367748 PMC3408070

[57] Darriba D, Taboada GL, Doallo R, Posada D. jModelTest 2: more models, new heuristics and parallel computing. Nature Methods. 2012; 9(8):772. 10.1038/nmeth.2109 PMC4594756 22847109

[58] Pfeiler E, Bitler BG, Ramsey JM, Palacios-Cardiel C, Markow TA. Genetic variation, population structure, and phylogenetic relationships of *Triatoma rubida* and *T*. *recurva* (Hemiptera: Reduviidae: Triatominae) from the Sonoran Desert, insect vectors of the Chagas’ disease parasite *Trypanosoma cruzi*. Molecular Phylogenetics and Evolution. 2006; 41(1):209–21. 10.1016/j.ympev.2006.07.001 16934496

[59] Ho SY, Phillips MJ, Cooper A, Drummond AJ. Time dependency of molecular rate estimates and systematic overestimation of recent divergence times. Molecular Biology and Evolution. 2005; 22(7):1561–8. 10.1093/molbev/msi145 15814826

[60] Rambaut A, Drummond AJ, Xie D, Baele G, Suchard MA. Posterior summarisation in Bayesian phylogenetics using Tracer 1.7. Systematic Biology. 2018. 10.1093/sysbio/syy032 PMC6101584 29718447

[61] Rambaut A. FigTree version 1.4. 2009; http://tree.bio.ed.ac.uk/software/figtree/.

[62] Benedetto JL. El continente de Gondwana a través del tiempo, una introducción a la geología histórica. Academia Nacional de Ciencias. Córdoba. Argentina. 2012.

[63] Clapperton CM. Nature of environmental changes in South America at the Last Glacial Maximum. Palaeogeography, Palaeoclimatology, Palaeoecology. 1993; 101(3–4):189–208.

[64] Haffer J. Speciation in amazonian forest birds. Science. 1969; 165(3889):131–7. 10.1126/science.165.3889.131 17834730

[65] Moreno-Mayar JV, Potter BA, Vinner L, Steinrucken M, Rasmussen S, Terhorst J, et al Terminal Pleistocene Alaskan genome reveals first founding population of Native Americans. Nature. 2018; 553(7687):203–7. 10.1038/nature25173 29323294

[66] Bush MB, Correa-Metrio A.Y., Hodell D.A., Brenner M., Anselmetti F.S., Ariztegui D. Re-evaluation of climate change in lowland Central America during the last glacial maximum using new sediment cores from Lake Petén-Itzá, Guatemala. 2009.

[67] Prentice IC, Harrison SP, Bartlein PJ. Global vegetation and terrestrial carbon cycle changes after the last ice age. The New Phytologist. 2011; 189(4):988–98. 10.1111/j.1469-8137.2010.03620.x 21288244

[68] Palacios D. The state of knowledge on the deglaciation of America in 2017. Geographical Research Letters. 2017; 43:361–76.

[69] Peterson AT, Soberón J, Sanchez-Cordero VV. Conservatism of ecological niches in evolutionary time. Science. 1999; 285(5431):1265–7. 10455053 10.1126/science.285.5431.1265

[70] Ramamoorthy TP, Bye R, Lot A, Fa J. Biological Diversity of Mexico Origins and Distribution. New York, Oxford Univ. Press 1993.

[71] Lowe A, Harris S, Ashton P. Ecological Genetics: Designs, Analysis, and Application. USA: Blackwell publishing 2004 320 p.

[72] Morales Gamboa A. Migraciones, regionalismo y ciudadanía en Centroamérica In: Villafuerte Solís D, García Aguilar MC, editors. Migraciones en el sur de México y Centroamérica. México: Universidad de Ciencias y Artes de Chiapas—Miguel Ángel Porrúa; 2008.

[73] Najera-Aguirre J. Conociendo la Encuesta sobre Migración en la Frontera Guatemala-México: alcances y limitaciones. población Pd, editor. 2010.

[74] Izeta-Alberdi A, Ibarra-Cerdeña CN, Pech-May A, Tun-Ku E, De la Cruz-Félix HK, López-Cancino SA, et al Phylogeography and lanscape genetics of *Trypanosoma cruzi* in the Mexican Neotropical region: are there any barriers to gene flow? Acta Tropica. 2018.

[75] Sasaki H, Rosales R, Tabaru Y. Host feeding profiles of *Rhodnius prolixus* and *Triatoma dimidiata* in Guatemala (Hemiptera: Reduviidae: Triatominae). Medical Entomology and Zoology. 2003; 54:283–9.

[76] Zeledon R, Solano G, Zuniga A, Swartzwelder JC. Biology and ethology of *Triatoma dimidiata* (Latreille, 1811). 3. Habitat and blood sources. Journal of Medical Entomology. 1973; 10(4):363–70. 4582846 10.1093/jmedent/10.4.363

[77] Cecere MC, Gurtler RE, Canale D, Chuit R, Cohen JE. The role of the peridomiciliary area in the elimination of *Triatoma infestans* from rural Argentine communities. Revista Panamericana de Salud Publica. 1997; 1(4):273–9. 9149523 10.1590/s1020-49891997000400003

[78] Pizarro JC, Stevens L. A new method for forensic DNA analysis of the blood meal in chagas disease vectors demonstrated using *Triatoma infestans* from Chuquisaca, Bolivia. PloS one. 2008; 3(10):e3585 10.1371/journal.pone.0003585 18974787 PMC2570791

[79] Gurtler RE, Cecere MC, Vazquez-Prokopec GM, Ceballos LA, Gurevitz JM, Fernandez Mdel P, et al Domestic animal hosts strongly influence human-feeding rates of the Chagas disease vector *Triatoma infestans* in Argentina. PLoS Neglected Tropical Diseases. 2014; 8(5):e2894 10.1371/journal.pntd.0002894 24852606 PMC4037315

[80] Izeta-Alberdi A, Tun-Ku E, Mazariegos-Hidalgo CJ, López-Cancino SA, Gutiérrez S, Albino-Miranda S, et al. Molecular marker sensitivity to diagnose and genotype Mexican Trypanosoma cruzi populations. Parasites & Vectors. 2018.

[81] Gurtler RE, Cardinal MV. Reservoir host competence and the role of domestic and commensal hosts in the transmission of *Trypanosoma cruzi*. Acta Tropica. 2015; 151:32–50. 10.1016/j.actatropica.2015.05.029 26051910

[82] Ramirez JD, Guhl F, Rendon LM, Rosas F, Marin-Neto JA, Morillo CA. Chagas cardiomyopathy manifestations and *Trypanosoma cruzi* genotypes circulating in chronic Chagasic patients. PLoS Neglected Tropical Diseases. 2010; 4(11):e899 Epub 2010/12/15. 10.1371/journal.pntd.0000899 21152056 PMC2994916

[83] Gurgel-Goncalves R, Komp E, Campbell LP, Khalighifar A, Mellenbruch J, Mendonca VJ, et al Automated identification of insect vectors of Chagas disease in Brazil and Mexico: the Virtual Vector Lab. PeerJ. 2017; 5:e3040 Epub 2017/04/26. 10.7717/peerj.3040 28439451 PMC5398287

