# Supplementary figures and images for: Genetic variation and phylogeography of the Triatoma dimidiata complex evidence a potential center of origin and recent divergence of haplogroups having differential Trypanosoma cruzi and DTU infections
Source: PLoS Negl Trop Dis. 2019 Jan 28;13(1):e0007044. doi: 10.1371/journal.pntd.0007044 (PMC6366694; doi:10.1371/journal.pntd.0007044)

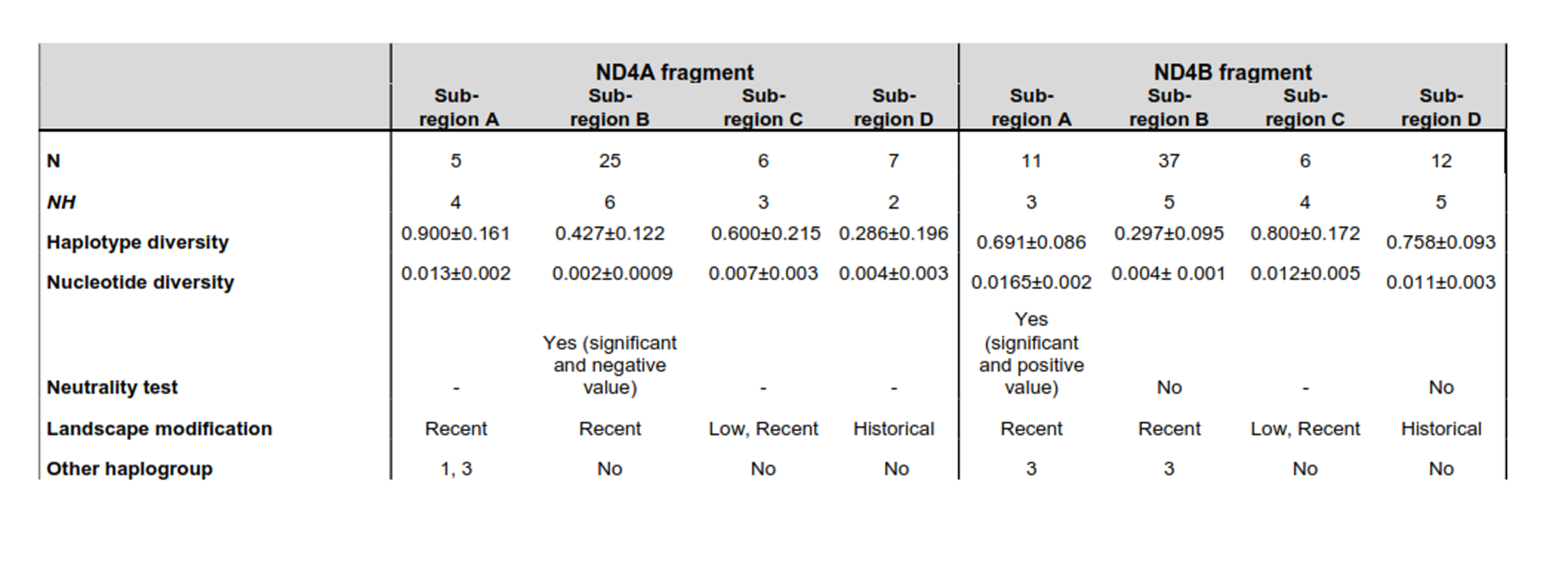

Supplement: S1 Fig — (TIF) [file pntd.0007044.s002.tif]

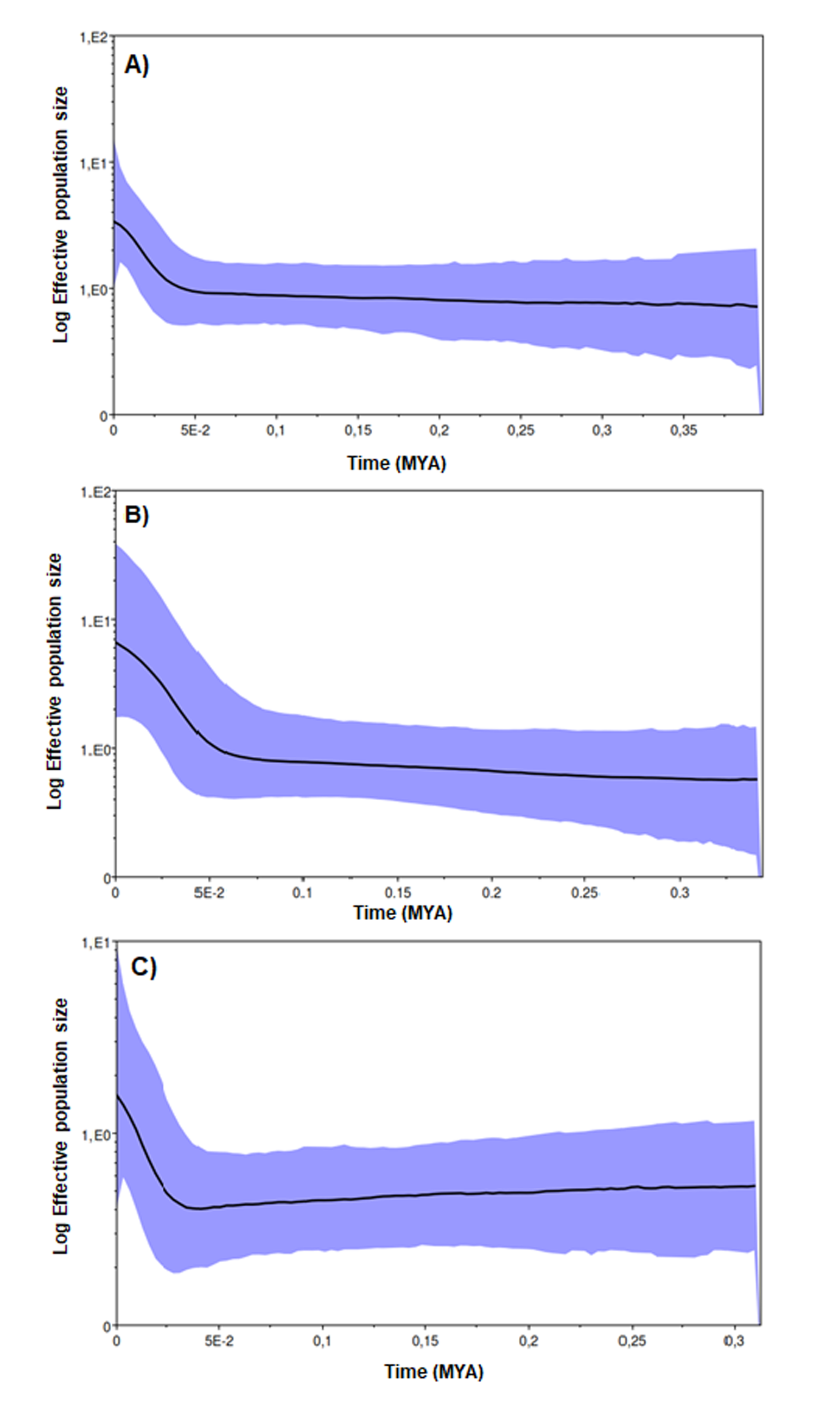

Supplement: S2 Fig — The relative population size measured as a product-effective population size (y-axis) is shown over time in millions of years (x-axis) in a simulated coalescent-based demographic model using standard Markov Chain Monte Carlo (MCMC). A) ND4A from Berriozábal + other Mexican Neotropical sites + continental GenBank sequences, B) ND4A from CA and Colombia only Hg3, C) ND4A from Berriozábal + other Mexican Neotropical sites. The thick black line is the median estimate and the solid (blue) interval shows the 95% highest posterior density limits. (TIF) [file pntd.0007044.s003.tif]
