# Supplementary material for: Genetic variation and phylogeography of the Triatoma dimidiata complex evidence a potential center of origin and recent divergence of haplogroups having differential Trypanosoma cruzi and DTU infections
Source: PLoS Negl Trop Dis. 2019 Jan 28;13(1):e0007044. doi: 10.1371/journal.pntd.0007044 (PMC6366694; doi:10.1371/journal.pntd.0007044)
Supplement: S1 File — (DOCX) [file pntd.0007044.s004.docx]

>H1 [organism=Triatoma dimidiata] Triatoma dimidiata haplotype H1 NADH dehydrogenase subunit 4 (ND4) gene for mitochondrial product

GCTGGTGTTCTTTTAAAGTTAGGGGGCTATGGTCTTATGCGTGTTTCTAATTTTATTTATGATTATTTATTTAAGTTTAGTTATGTTTTTGTTGGTTTAAGACTTTATGGTGCTTTTTTGGTCGGGTTTTTATGTTTATATCAGATTGATATTAAGTC

>H2 [organism=Triatoma dimidiata] Triatoma dimidiata haplotype H2 NADH dehydrogenase subunit 4 (ND4) gene for mitochondrial product

GCTGGTGTTCTTTTAAAGTTAGGGGGCTATGGTCTTATGCGTGTTTCTAATTTTATTTATGATTATTTATTTAAGTTTAGTTACGTTTTTGTTGGTTTAAGACTTTATGGTGCTTTTTTGGTCGGGTTTTTATGTTTATATCAGATTGATATTAAGTC

>H3 [organism=Triatoma dimidiata] Triatoma dimidiata haplotype H3 NADH dehydrogenase subunit 4 (ND4) gene for mitochondrial product

GCTGGTGTTCTTTTAAAGTTAGGGGGCTATGGTCTTATGCGTGTTTCTAATTTTATTTATGATTATTTATTTAAGTTTAGTTATGTTTTTGTTGGTTTAAGACTTTATGGTGCTTTTTTGGTCGGGTTTTTATGTTTATTTCAGATTGATATTAAGTC

>H4 [organism=Triatoma dimidiata] Triatoma dimidiata haplotype H4 NADH dehydrogenase subunit 4 (ND4) gene for mitochondrial product

GCTGGTGTTCTTTTAAAGTTAGGGGGTTATGGTCTTATGCGTGTTTCTAATTTTATTTATGATTATTTATTTAAGTTTAGTTATGTTTTTGTTGGTTTAAGACTTTATGGTGCTTTTTTGGTCGGGTTTTTATGTTTATATCAGATTGATATTAAGTC

>H5 [organism=Triatoma dimidiata] Triatoma dimidiata haplotype H5 NADH dehydrogenase subunit 4 (ND4) gene for mitochondrial product

GCTGGTGTTCTTTTAAAGTTAGGGGGTTATGGTCTTATGCGTGTTTCTAATTTTATTTATGATTATTTATTTAAGTTTAGTTATGTTTTTGTTGGTTTAAGACTTTATGGTGCTTTTTTGGTCGGGTTTTTATGTTTATATCAGATTGATATCAAGTC

>H6 [organism=Triatoma dimidiata] Triatoma dimidiata haplotype H6 NADH dehydrogenase subunit 4 (ND4) gene for mitochondrial product

GCTGGTGTTCTTTTAAAGTTAGGGGGTTATGGTCTTATGCGTGTTTCTAATTTTATTTATGATTATTTATTTAAGTTTAGTTATGTTTTTGTTGGTTTAAGACTTTATGGTGCTTTTTTGGTCGGGTTTTTATGTTTATATCAGAATGATATTAAGTC

>H7 [organism=Triatoma dimidiata] Triatoma dimidiata haplotype H7 NADH dehydrogenase subunit 4 (ND4) gene for mitochondrial product

GCTGGTGTTCTTTTAAAGTTAGGGGGCTATGGTCTTATGCGTGTTTCTAATTTTATTTATGATTATTTATTTAAGTTTAGTTATGTTTTTGTTGGTTTAAGACTTTATGGTGCTTTTTTGGTCGGGTTTTTATGTTTATATCAAATTGATATTAAGTC

>H8 [organism=Triatoma dimidiata] Triatoma dimidiata haplotype H8 NADH dehydrogenase subunit 4 (ND4) gene for mitochondrial product

GCTGGTGTTCTTTTAAAGTTAGGGGGTTATGGTCTTATGCGTGTTTCTAATTTTATTTATGATTATTTATTTAAGTTTAGTTATGTTTTTGTTGGTTTAAGACTTTATGGTGCTTTTTTGGTTGGGTTTTTATGTTTATATCAGATTGATATTAAGTC

>H9 [organism=Triatoma dimidiata] Triatoma dimidiata haplotype H9 NADH dehydrogenase subunit 4 (ND4) gene for mitochondrial product

GCTGGTGTTCTTTTAAAGTTAGGGGGTTATGGTCTTATGCGTGTTTCTAATTTTATTTATGATTATTTATTTAAGTTTAGTTATGTTTTTGTTGGTTTAAGACTTTATGGTGCTTTTTTAGTTGGGCTTTTATGTTTATATCAGATTGATATTAAGTC

>H10 [organism=Triatoma dimidiata] Triatoma dimidiata haplotype H10 NADH dehydrogenase subunit 4 (ND4) gene for mitochondrial product

GCTGGTGTTCTTTTAAAGTTAGGGGGCTATGGTCTTATGCGTGTTTCTAATTTTATTTATGGTTATTTGTTTAAGTTTGGTTATGTTTTTATTGGTTTAAGACTTTATGGTGCTTTTTTGGTTGGGTTTTTATGTTTATATCAGGTTGATATTAAATC

>H11 [organism=Triatoma dimidiata] Triatoma dimidiata haplotype H11 NADH dehydrogenase subunit 4 (ND4) gene for mitochondrial product

GCTGGTGTTCTTTTAAAGTTAGGGGGTTATGGTCTTATACGTGTTTCTAATTTTATTTATAGTTATTTATTTAATTTTGGTTATGTTTTTATTGGTTTAAGACTTTATGGTGCTTTTTTGGTTGGGTTTTTATGTTTATATCAGGTTGATATTAAATC

>H12 [organism=Triatoma dimidiata] Triatoma dimidiata haplotype H12 NADH dehydrogenase subunit 4 (ND4) gene for mitochondrial product

GCTGGTGTTCTTTTAAAGTTAGGGGGTTATGGTCTTATACGTGTTTCTAATTTTATTTATGGTTATTTGTTTAAGTTTGGTTATGTTTTTATTGGTTTAAGACTTTATGGTGCTTTTTTGGTTGGGTTTTTATGTTTATATCAGGTTGATATTAAATC

>H13 [organism=Triatoma dimidiata] Triatoma dimidiata haplotype H13 NADH dehydrogenase subunit 4 (ND4) gene for mitochondrial product

GCTGGTGTTCTTTTAAAGTTAGGGGGTTATGGTCTTATACGTGTTTCTAATTTTATTTTTAGTTATTTATTTAATTTTGGTTATGTTTTTATTGGTTTAAGACTTTATGGTGCTTTTTTGGTTGGGTTTTTATGTTTATATCAGGTTGATATTAAATC

>H14 [organism=Triatoma dimidiata] Triatoma dimidiata haplotype H14 NADH dehydrogenase subunit 4 (ND4) gene for mitochondrial product

GCTGGTGTTCTTTTAAAGTTAGGGGGTTATGGTCTTATACCTGTTTCTAATTTTATTTATAGTTATTTATTTAATTTTGGTTATGTTTTTATTGGTTTAAGACCTTATGGTGCTTTTTTGGTTGGGTTTTTATGTTTATATCCAGTTGATATTAAATC

>H15 [organism=Triatoma dimidiata] Triatoma dimidiata haplotype H15 NADH dehydrogenase subunit 4 (ND4) gene for mitochondrial product

GCTGGTGTTCTTTTAAAGTTAGGGGGTTATGGTCTTATACGTGTTTCTAATTTTATTTATAGTTATTTATTTAATTTTGGTTATGTTTTTATTGGTTTAAGACTTTATGGTGCTTTTTTGGTTGGGTTTTTATGTTTATATCAAGTTGATATTAAATC

>H16 [organism=Triatoma dimidiata] Triatoma dimidiata haplotype H16 NADH dehydrogenase subunit 4 (ND4) gene for mitochondrial product

GCTGGTGTTCTTTTAAAGTTAGGGGGTTATGGTCTTATACCTGTTTCTAATTTTATTTATAGTTATTTATTTAATTTTGGTTATGTTTTTATTGTTTTAAGACTTTATAGTGCTTTTTTGGTTGGGTTTTTATGTTTATATCAAGTTGATATTAAATC

>H17 [organism=Triatoma dimidiata] Triatoma dimidiata haplotype H17 NADH dehydrogenase subunit 4 (ND4) gene for mitochondrial product

GCTGGTGTTCTTTTAAAGTTAGGGGGTTATGGTCTTATACGTGTTTCTAATTTTATTTATAGTTATTTATTTAATTTTGGTTATGTTTTTATTGGTTTAATACTTTATGGTGCTTTTTTGGTTGGGTTTTTATGTTTATATCAGGTTGATATTAAATC

>H18 [organism=Triatoma dimidiata] Triatoma dimidiata haplotype H18 NADH dehydrogenase subunit 4 (ND4) gene for mitochondrial product

GCTGGTGTTCTTTTAAAGTTAGGGGGTTATGGTCTTATACGTGTTTCTAATTTTATTTATAGTTATTTGTTTAATTTTGGTTATGTTTTTATTGGTTTAAGACTTTATGGTGCTTTTTTGGTTGGGTTTTTATGTTTATATCAGGTTGATATTAAATC

>H19 [organism=Triatoma dimidiata] Triatoma dimidiata haplotype H19 NADH dehydrogenase subunit 4 (ND4) gene for mitochondrial product

GCTGGTGTTCTTTTAAAGTTAGGGGGTTATGGCCTTATACGTGTTTCTAATTTTATTTATAGTTATTTATTTAATTTTGGTTATGTTTTTATTGGTTTAAGACTTTATGGTGCTTTTTTGGTTGGGTTTTTATGTTTATATCAGGTTGATATTAAATC

>H20 [organism=Triatoma dimidiata] Triatoma dimidiata haplotype H20 NADH dehydrogenase subunit 4 (ND4) gene for mitochondrial product

GCTGGTGTTCTTTTAAAGTTAGGGGGCTATGGTCTTATACGTGTTTCTAATTTTATTTATGGTTATTTGTTTAAGTTTGGTTATGTTTTTATTGGTTTAAGACTTTATGGTGCTTTTTTGGTTGGGTTTTTATGTTTATATCAGGTTGATATTAAATC

>H21 [organism=Triatoma dimidiata] Triatoma dimidiata haplotype H21 NADH dehydrogenase subunit 4 (ND4) gene for mitochondrial product

GCTGGTGTTCTTTTAAAGTTAGGGGGTTATGGTTTTATACGTGTTTCTAATTTTATTTATGGTTATTTGTTTAAGTTTGGTTATGTTTTTATTGGTTTAAGACTTTATGGTGCTTTTTTGGTTGGGTTTTTATGTTTATATCAGGTTGATATTAAATC

>H22 [organism=Triatoma dimidiata] Triatoma dimidiata haplotype H22 NADH dehydrogenase subunit 4 (ND4) gene for mitochondrial product

GCTGGTGTTCTTTTAAAGTTAGGGGGTTATGGTCTTATACGTGTTTCTAATTTTATTTATGGCTATTTGTTTAAGTTTGGTTATGTTTTTATTGGTTTAAGACTTTATGGTGCTTTTTTGGTTGGGTTTTTATGTTTATATCAGGTTGATATTAAATC

>H23 [organism=Triatoma dimidiata] Triatoma dimidiata haplotype H23 NADH dehydrogenase subunit 4 (ND4) gene for mitochondrial product

GCTGGTGTTCTTTTAAAGTTAGGGGGCTATGGTCTTATACGTGTTTCTAATTTTATTTATGGTTATTTGTTTAAGTTTGGTTATGTTTTTATTGGTTTAAGACTTTATGGTGCTTTTTTGTTTGGGTTTTTATGTTTATATCAGGTTGATTTTAAATC

>H24 [organism=Triatoma dimidiata] Triatoma dimidiata haplotype H24 NADH dehydrogenase subunit 4 (ND4) gene for mitochondrial product

GCTGGTGTTCTTTTAAAGTTAGGGGGCTATGGTCTTATACGTGTTTCTAATTTTATTTATGGTTATTTGTTCAAGTTTGGTTATGTTTTTATTGGTTTAAGACTTTATGGTGCTTTTTTAGTTGGGTTTTTATGTTTATATCAGGTTGATATTAAGTC

>H25 [organism=Triatoma dimidiata] Triatoma dimidiata haplotype H25 NADH dehydrogenase subunit 4 (ND4) gene for mitochondrial product

GCTGGTGTTCTTTTAAAGTTAGGGGGCTATGGTCTTATACGTGTTTCTAATTTTATTTATGGCTATTTGTTCAAGTTTGGTTATGTTTTTATTGGTTTAAGACTTTATGGTGCTTTTTTAGTTGGGTTTTTATGTTTATATCAGGTTGATATCAAGTC

>H26 [organism=Triatoma dimidiata] Triatoma dimidiata haplotype H26 NADH dehydrogenase subunit 4 (ND4) gene for mitochondrial product

GCTGGTGTTCTTTTAAAGTTAGGGGGCTATGGTCTTATACGTGTTTCTAATTTTATTTATGGTTATTTGTTTAAGTTTGGTTATGTTTTTATTGGTTTAAGACTTTATGGTGCTTTTTTAGTTGGGTTTTTATGTTTATATCAGGTTGATATTAAATC

>H27 [organism=Triatoma dimidiata] Triatoma dimidiata haplotype H27 NADH dehydrogenase subunit 4 (ND4) gene for mitochondrial product

GCTGGTGTTCTTTTAAAGTTAGGGGGCTATGGTCTTATACGTGTTTCTAATTTTATTTATGATTATTTGTTCAAGTTTGGTTATGTTTTTATTGGTTTAAGACTTTATGGTGCTTTTTTAGTTGGGTTTTTATGTTTATATCAGGTTGATATTAAGTC

>H28 [organism=Triatoma dimidiata] Triatoma dimidiata haplotype H28 NADH dehydrogenase subunit 4 (ND4) gene for mitochondrial product

GCTGGTGTCCTTTTAAAGTTAGGGGGTTATGGTTTTATACGTGTTTCTAATTTTATTTGTGATTATTTGTTTAAATTTGGTTATGTTTTTATTGGTTTGAGACTTTATGGTGCTTTTTTAGTTGGGTTTTTATGTTTGTATCAGGTTGATATTAAATC

>H29 [organism=Triatoma dimidiata] Triatoma dimidiata haplotype H29 NADH dehydrogenase subunit 4 (ND4) gene for mitochondrial product

GCTGGTGTCCTTTTAAAGTTAGGGGGTTATGGTCTTATACGTGTTTCTAATTTTATTTGTGATTATTTGTTTAAATTTGGTTATGTTTTTATTGGTTTGAGACTTTATGGTGCTTTTTTAGTTGGGTTTTTATGTTTGTATCAGGTTGATATTAAATC

>H30 [organism=Triatoma dimidiata] Triatoma dimidiata haplotype H30 NADH dehydrogenase subunit 4 (ND4) gene for mitochondrial product

GCTGGTGTTCTTCTGAAGTTAGGGGGCTATGGTCTTATACGTGTTTCTAATTTTATTTGTGATTATTTGTTTAAATTTGGTTATGTTTTTATTGGTTTGAGACTTTATGGTGCTTTTTTAGTTGGGCTTTTATGTTTGTATCAGGTTGATATTAAGTC

>H31 [organism=Triatoma dimidiata] Triatoma dimidiata haplotype H31 NADH dehydrogenase subunit 4 (ND4) gene for mitochondrial product

GCTGGTGTCCTTCTGAAGTTAGGGGGCTATGGTCTTATACGTGTTTCTAATTTTATTTGTGATTATTTGTTTAAATTTGGTTATGTTTTTATTGGTTTGAGACTTTATGGTGCTTTTTTAGTTGGGCTTTTATGTTTGTATCAGGTTGATATTAAGTC

>H32 [organism=Triatoma dimidiata] Triatoma dimidiata haplotype H32 NADH dehydrogenase subunit 4 (ND4) gene for mitochondrial product

GCTGGTGTCCTTTTGAGGTTGGGGGGTTACGGTCTTATGCGTGTTTCTAATTTTATTTGTGACTATTTGTTTGGTTTTGGTTATGTTTTTATTGGTTTGAGACTTTATGGTTCTTTTTTAGTTGGGCTTTTATGTTTGTATCAGGTTGATATTAAATC

>H33 [organism=Triatoma dimidiata] Triatoma dimidiata haplotype H3 NADH dehydrogenase subunit 4 (ND4) gene for mitochondrial product

GATGGTGTCCTTTTGAGGTTGGGGGGTTACGGTATTATGCGTGTTTCTAATTTTATTTGTGACTATTTGTTTGGTTTTGGTTATGTTTTTATTGGTTTGAGACTTTATGGTTCTTTTTTAGTTGGGCTTTTATGTTTGTATCAGGTTGATATTAAATC

>H1 [organism=Triatoma pallidipennis] Triatoma pallidipennis haplotype H1 NADH dehydrogenase subunit 4 (ND4) gene for mitochondrial product

GCTGGGGTTTTATTAAAGTTGGGGGGTTATGGTCTTATGCGTGTTTCTAATTTTATTTATGATTATTTGTTTAAGTATAGTTATGTTTTTGTTGGTTTGAGACTTTATGGTGCTTTTTTGGTTGGGTTTTTTTGTTTGTATCAAATTGATATTAAATC
